# Geochemical processes controlling the groundwater chemistry and fluoride contamination in the Yuncheng Basin, China—An area with complex hydrogeochemical conditions

**DOI:** 10.1371/journal.pone.0199082

**Published:** 2018-07-26

**Authors:** Wenting Luo, Xubo Gao, Xin Zhang

**Affiliations:** School of Environmental Studies, China University of Geosciences, Wuhan, China; University of Pittsburgh, UNITED STATES

## Abstract

Hydrogeochemical and stable isotope analyses and geochemical modeling were carried out to identify the major geochemical processes controlling the groundwater chemistry and fluoride contamination in the aquifers of the Yuncheng Basin, China, an area with complex hydrogeochemical conditions and severe fluoride contamination of the groundwater. The major findings of this case study include the following: 1) Cation exchange and salt effects are vital controls on the enrichment of fluoride in groundwater in the area by reducing the activity of Ca^2+^/F^-^ in groundwater via ion complexation. Cation exchange increased the fluoride concentration by 2.7 mg/L when the Na/Ca molar ratio increased from 0.24 to 9.0, while the salt effect led to a ca. 5–10% increase in complex F^-^ in groundwater due to the further dissolution of fluoride-bearing minerals in the aquifers, as suggested by a model calculation. 2) Anthropogenic contamination from pesticide and fertilizer use and industrial waste discharge is also a main source of fluoride in the groundwater. 3) Evaporation and ion effects favor the enrichment of fluoride in groundwater by encouraging the removal of Ca via precipitation. 4) The desorption of fluoride from mineral/organic matter surfaces is enhanced under alkaline conditions and a high HCO_3_ content in groundwater.

## Introduction

Groundwater is becoming increasingly important for the drinking water supply, ecosystem health and economic development as the global population grows. Groundwater is fairly ubiquitous, but its conditions vary enormously, and exploitation is often undertaken with a limited understanding of the hydrochemistry and without sufficient evaluation of the resource quality, especially in developing countries. Potentially toxic elements, e.g., fluorine, may reach hazardous concentrations in groundwater as a result of specific hydrogeochemical processes and contamination due to human activities. Elevated concentrations of fluoride in groundwater that exceeded the World Health Organization (WHO) limit of 1.5 mg/L in drinking water have been detected in many parts of the world [1–12]. The mechanisms of fluoride contamination in groundwater systems in arid-semi arid regions, where groundwater may be the only or most important water supply source, are of scientific, environmental and ecological importance.

The dissolution of natural fluoride-bearing minerals, such as fluorspar, fluorapatite, amphiboles, hornblende, tremolite and biotite, is the common natural source of fluoride in groundwater [13–23]. Ion exchange, evaporation, adsorption-desorption, ion competition, mixing, salinization and anthropogenic pollution are geochemical processes that can form fluoride-rich groundwater. However, the sources and processes of fluoride contamination can vary with the geochemical and hydrochemical conditions [24–28]; thus, studies of the hydrogeochemical processes in areas with groundwater highly contaminated by fluoride are important for understanding the mechanisms involved. Most previous studies have focused on the hydrogeochemical processes of fluoride in aquifers [29–34], while the identification of these processes remains challenging in areas with complex hydrogeochemical or geo-hydrogeological conditions.

The Yuncheng Basin, which is located in North China, contains severely fluoride-polluted groundwater and has complicated hydrogeochemical conditions [5],[17],[35–38], was selected for this study. Hydrogeochemistry and stable isotope analyses and geochemical modeling were employed to understand the sources and mechanisms of fluoride contamination in this specific groundwater system. The aims of this study were (1) to investigate the major geochemical processes controlling the groundwater chemistry in the Yuncheng Basin and (2) to identify the mechanisms responsible for the fluoride contamination of the groundwater.

## Hydrogeology

**Fig 1:**
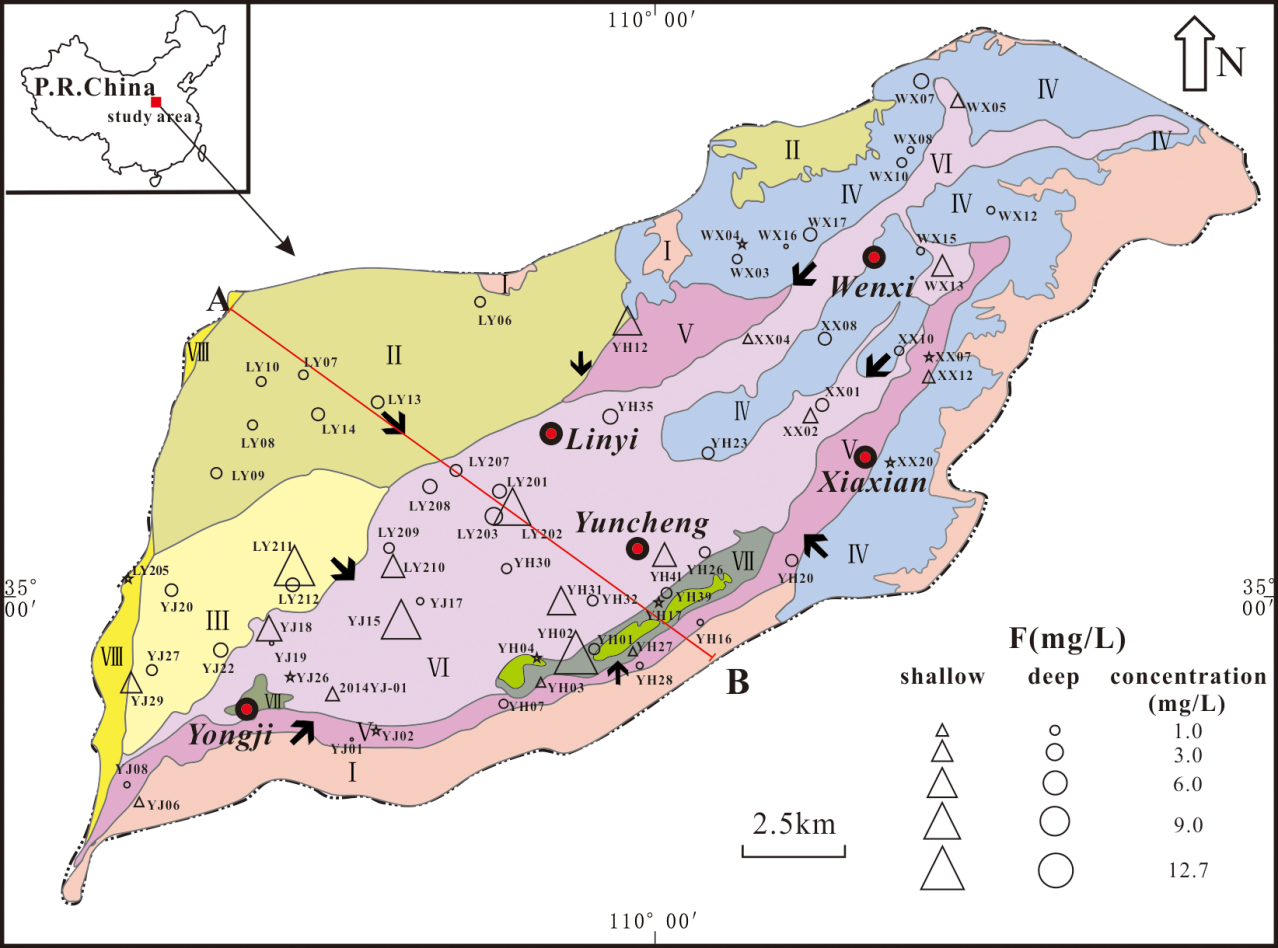
Simple geomorphological map of the Yuncheng Basin, China. (Areas: I, mountain bedrock areas; II, Emei high platform; III, Kaolao low platform; IV, loess hilly regions; V, piedmont plain; VI, alluvial plain; VII, fluvial depressions; VIII, Yellow River terrace). → groundwater flow direction; pentagram, surface water; triangle, shallow groundwater; circle, deep groundwater; Line AB, location of cross-section in Fig 2.

The Yuncheng Basin is situated in the southwestern region of Shanxi Province, with hills surrounding it in three directions—north, east and south (Fig 1). The basin consists of several aquifers and contains mainly thick, loose, Quaternary sediment rich in pores. The sediment consists of mainly fine to coarse sand, gravel, pebbles, silt and clay and has a maximum thickness of approximately five hundred meters.

Phreatic and artesian aquifers are the main water resources for exploitation in the study area. Based on the distribution characteristics of the aquifers and the hydraulic features, Quaternary groundwaters in the Yuncheng Basin were classified into two types in this study: (1) phreatic aquifers (5–70 m), which are supplied by precipitation, canal seepage, irrigation infiltration, reservoir seepage and lateral recharge and have discharge that is dominated by artificial exploitation and, in some parts, evaporation, and (2) semi-confined/confined aquifers (85–500 m), which are remotely recharged mainly by lateral runoff from mountain fronts and leakage through aquitards and have discharge that is dominated by artificial exploitation. For detailed geological and hydrological information, see Gao et al. [37] and Li et al. [38].

## Sampling and methods

A total of seventy samples, including one precipitation, eight surface-water, nineteen shallow-aquifer, and 42 deep-aquifer samples, were collected from different areas of the Yuncheng Basin during August, 2013-September, 2015. Water samples were collected after the in situ physicochemical parameters, including temperature, pH and electrical conductivity (EC), were stable; all the parameters were measured by portable meters.

Each sample was collected in three bottles—one for anion analysis, one with added acid for cation analysis and one stable isotope determination. The total alkalinity was measured on the sampling day using the Gran titration method with an error of < ± 2% for triplicate analyses. The concentrations of F^-^, Cl^−^, SO_4_^2−^, and NO_3_^−^ were determined using ion chromatography (IC, Dionex 120, Dionex, Sunnyvale, CA, USA). The Dionex IonPac AS19-4μm column meets the performance requirements specified in U.S. EPA Methods 300.0 and 300.1 for the determination of anion in groundwater. Certified anion standard solutions (1000 μg/mL, National Institute of Metrology) and deionized water were prepared for the composite working standards. For cation analysis, reagent-grade HNO_3_ was added until the pH of samples was less than 2. Major cations, K^+^, Na^+^, Ca^2+^ and Mg^2+^, were measured using inductively coupled plasma-atomic emission spectrometry (ICP-AES, IRIS Intrepid XSP, Thermo Elemental, Madison, WI, USA) with an analytical error of less than ± 2%. For water samples, the analytical charge imbalances were within the standard limit of ±5%.

Oxygen-18 and deuterium analyses were performed in the laboratory of the Institute of Karst Geology at the Chinese Academy of Geological Sciences. The δ^18^O values of the water samples were determined by the CO_2_-H_2_O equilibration method [39] and measured using a gas source mass spectrometer (MAT 253). The δD values of the water samples were obtained by reduction to H_2_ over hot metallic zinc, as described by Coleman et al. [40], and measured using a gas source mass spectrometer (MAT 253). The isotopic data are reported in the standard delta notation in parts per thousand relative to Vienna Standard Mean Ocean Water (VSMOW) [41], and the δ^18^O and δD measurements had an overall precision of 0.2 and 2 ‰, respectively.

The mineral compositions of the sediment and base rock samples were analyzed using X-ray diffraction (XRD, ZSX Primus II). Semi-quantitative XRD was carried out by using the reference internal standard method [42] with an analytical error of ±10% for major minerals and ±30% for minor minerals. Scanning electron microscopy (SEM, Hitachi SU8010) was coupled with focused energy dispersive X-ray analysis for elemental semi-quantification. The samples were prepared in graphite stubs and coated with gold before analysis. The detection limit of the microprobe analyses was approximately 2.0% for fluorine.

To determine the contents of water-soluble fluoride and total fluorine, pesticides and fertilizers were first dissolved in DI water. In this study, the pesticides and fertilizers were all water soluble, so the water-soluble fluoride and total fluorine contents were the same. The total fluorine in the solid waste samples was analyzed by the method suggested by Gao et al. [43]. Water-soluble fluoride in the solid wastes was extracted by using DI water at a solid:liquid ratio of 1:10 (1 g of solid in 10 mL of DI water) at 25 °C after allowing a half hour for equilibration. The fluorine concentration in the equilibrated solution was further analyzed by IC (Dionex 120, Dionex, Sunnyvale, CA, USA). The analytical error of the total fluorine and water-soluble fluoride measurements was less than ±10%. Geochemical modeling and calculation of the saturation index (SI) of the minerals and fluoride species were performed with PHREEQC software.

The authors state that no specific permissions were required for the locations/activities in this research. This research is based on the National Natural Science Foundation of China (41372251). The Yuncheng basin was chosen as the study area and this was clearly stated in the project. So the specific permissions were not required. The authors state that the field studies did not involve any endangered or protected species.

## Results and discussion

### Complex hydrochemical conditions in the area

**Fig 2:**
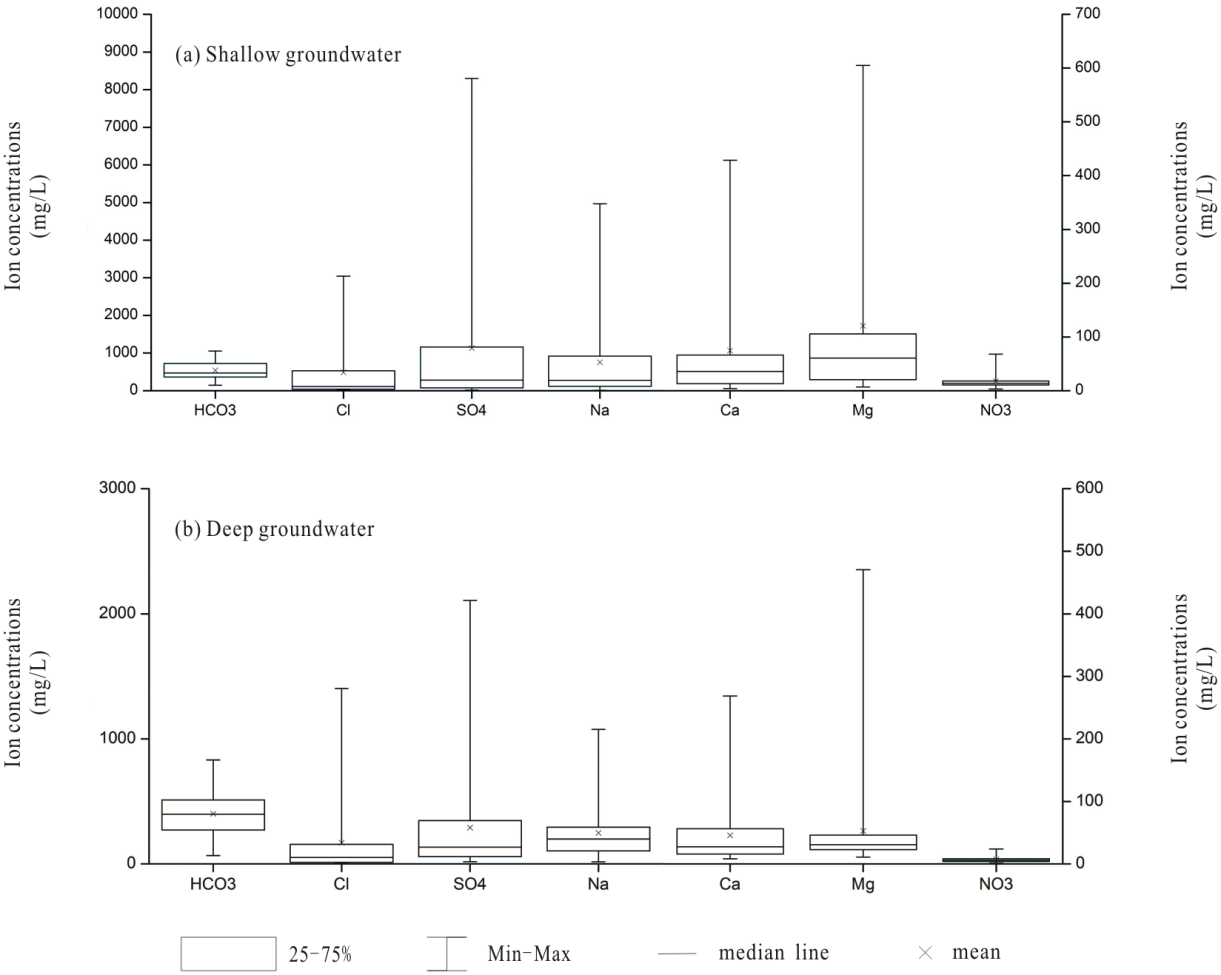
Box plots of the groundwater concentrations of the major ions in the Yuncheng Basin. (a), shallow groundwater; (b), deep groundwater. The concentrations of Ca, Mg and NO_3_^-^ refer to the right axis.

Groundwater samples were divided into two categories, shallow groundwater and semi-confined/confined deep groundwater, based on the burial depth and regional hydrogeological conditions. The major properties and the hydrochemistry of the shallow and deep groundwater are varied in the study area (Table 1, Fig 2). The concentration of sulfate (SO_4_^2-^), which ranged between 17.5 and 8,295 mg/L, was the most variable of the anions, followed by Cl^-^ (6.73–3,044 mg/L) and HCO_3_^-^ (66.45–1,053 mg/L) (Fig 2). The concentration of Na^+^, the major dominant cation, varied over a wide range of 8.28–4,967 mg/L, while the concentrations of other cations, including Ca^2+^, Mg^2+^, and K^+^, were relatively stable.

**Table 1:** Major physicochemical properties of water samples taken in the Yucheng Basin. (units, mg/L, except for the well depth, temperature, pH and isotope data).

Water Type		Well Depth(m)	Temperature (°C)	pH	HCO_3_	F	Cl	SO_4_	NO_3_	Ca	Mg	K	Na	TDS	δ^18^O	δD
**Rain water**			18.2	6.24	72	0.32	3.97	5.25	1.17	3.2	db	0.42	24.6	74.5	-	-
**Surface water (n = 8)**	Max.	-	29.0	9.14	1,939	15.36	52,598	34,487	163.6	163.1	765	31.20	49448	137,849	-48.0	-5.22
	Min.	-	5.1	7.75	181.9	0.32	10.57	70.72	1.39	24.04	3.12	2.24	17.56	289	-64.1	-7.68
	Mean	-	20.2	8.25	561.7	5.01	7,506	5,887	33.87	71.37	217.0	10.84	7,413	21,409	-57.07	-6.75
	Median	-	21.8	8.21	326.3	2.51	154	196	9.21	60.92	44.04	5.27	187	831	-59.1	-7.34
	SD.	-	7.0	0.46	580.4	5.72	18294	11,881	55.64	46.14	284.9	10.67	17,133	47,579	8.24	1.33
**Shallow groundwater (n = 19)**	Max.	70	23.4	8.6	1,053	12.65	3044	8295	67.88	428.9	605.2	14.08	4,967	17,452	-53.0	-6.57
	Min.	9	15.8	7.1	148.3	0.53	10.86	18.51	3.1	4	6.96	0.30	8.28	362.4	-67.9	-9.40
	Mean	39	18.0	7.8	539.6	4.18	489.9	1130	17.67	74.71	120.48	2.65	752.8	2,857	-62.4	-8.24
	Median	35	17.8	7.8	467.9	3.06	110.9	279.8	13.88	35.87	60.72	1.75	275.8	951.2	-62.7	-8.32
	SD.	20.2	1.62	0.4	246.5	3.92	804.0	2065	14.97	107.4	169.6	3.14	1,172	4,263	4.28	0.81
**Deep groundwater (n = 42)**	Max.	350	24.7	8.6	830.9	3.15	1,402	2,106	23.96	268.5	470.4	10.05	1,074	5,254	-62.9	-8.33
	Min.	85	16.5	7.3	66.4	0.10	6.73	17.51	1.17	8.21	10.92	0.971	16.25	228.8	-78.6	-10.84
	Mean	203	20.2	8.0	399.2	1.23	145.4	286.2	7.02	44.23	53.76	3.10	230.2	969.5	-71.2	-9.67
	Median	200	19.8	8.1	371.8	1.18	50.31	130.4	5.8	26.95	31.74	2.46	198.1	643.3	-69.65	-9.79
	SD.	73.3	2.03	0.3	164.7	0.66	279.3	449.0	4.64	50.59	79.14	2.19	208.1	1,034	5.93	0.86

Db, below detection limit; SD, the standard deviation.

**Fig 3:**
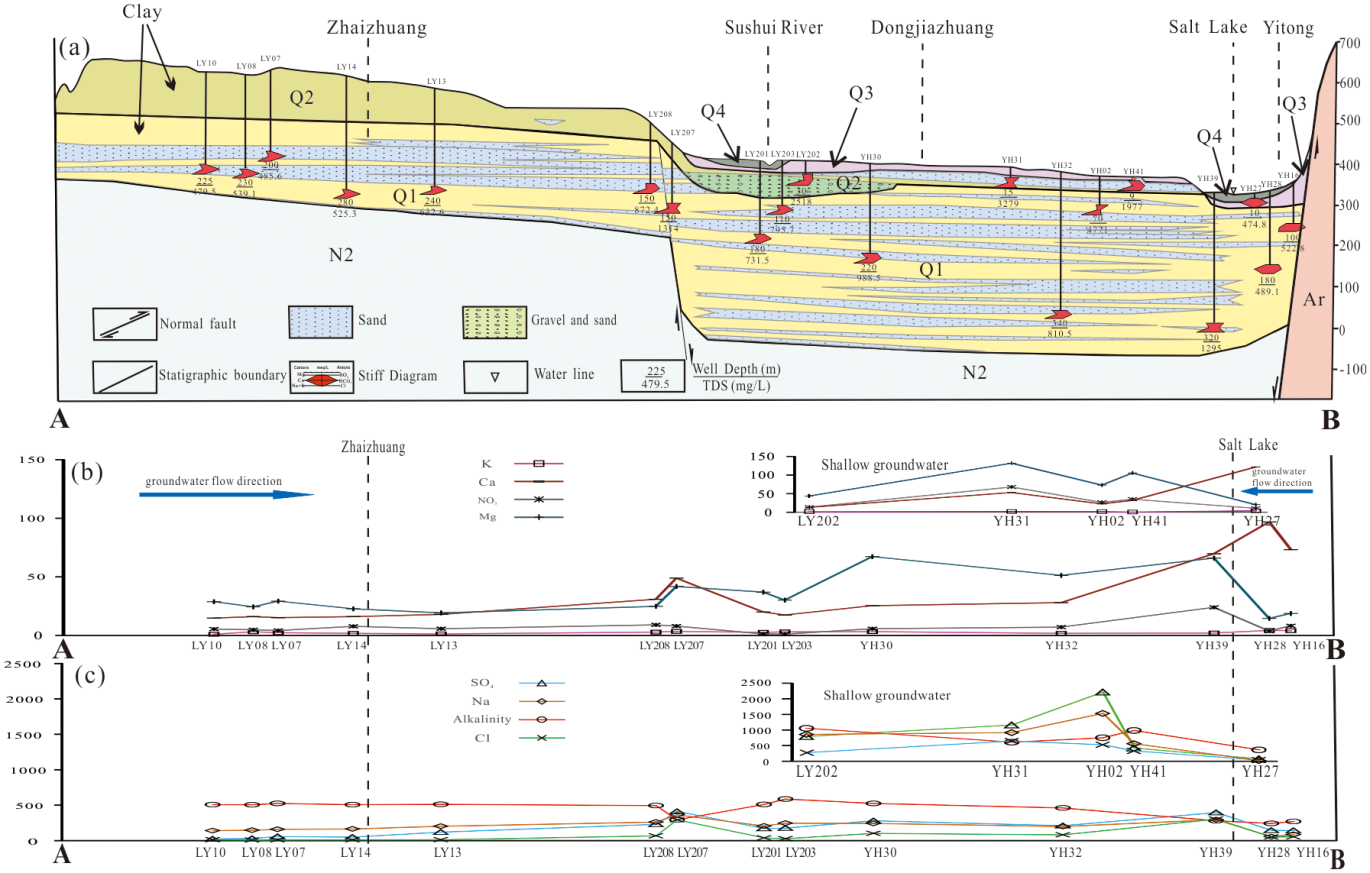
Schematic diagram of the distribution of major aquifers, hydrochemical facies and ion concentrations in groundwater. Q1, Lower Pleistocene; Q2, Middle Pleistocene; Q3, Upper Pleistocene.

**Fig 4:**
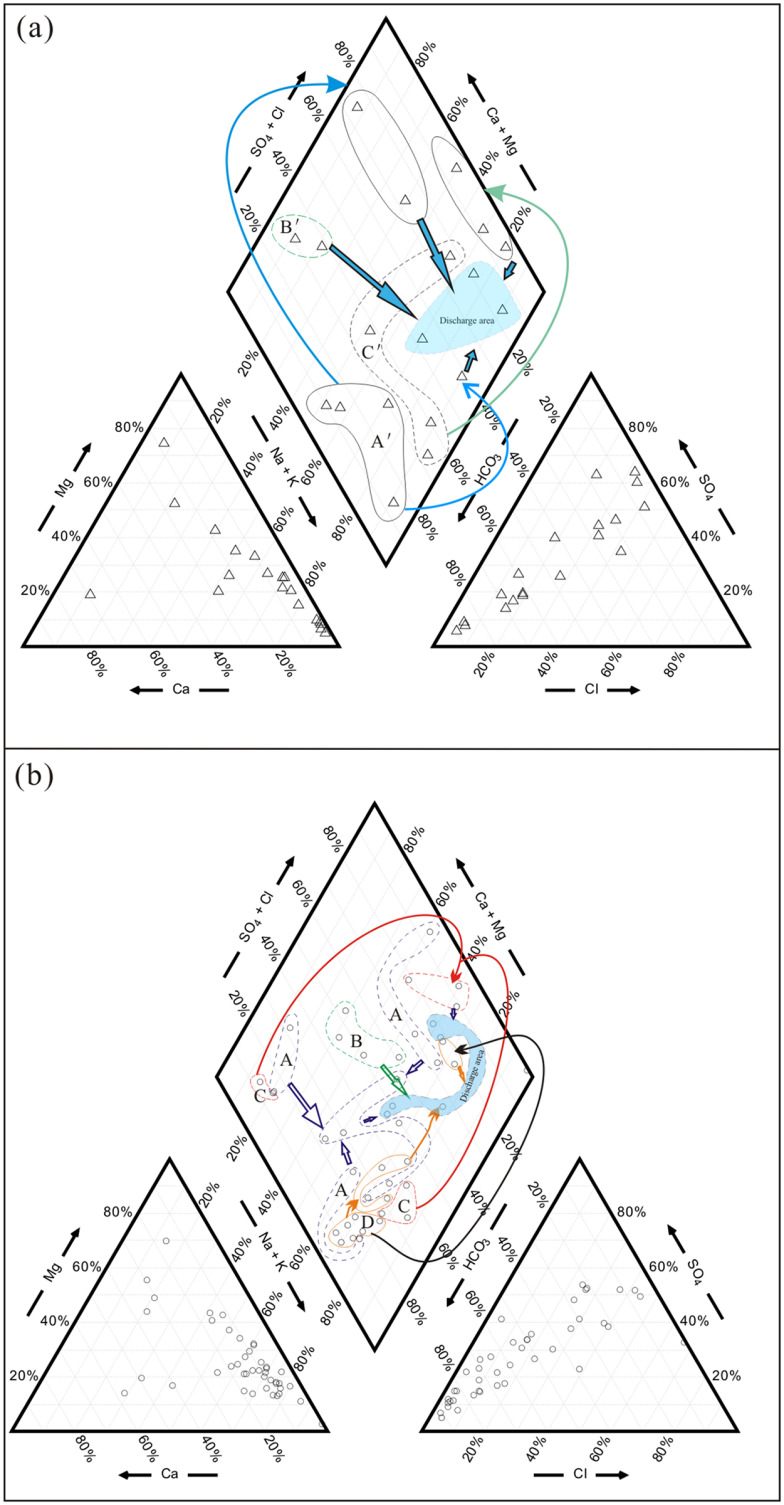
Piper diagrams of groundwater samples from the Yuncheng Basin. Subgroups: (a) shallow groundwater—A′, samples from the northeastern areas; B′, samples from the southern mountain areas; and C′, samples from the western areas; (b) deep groundwater—A, samples from the northeastern areas; B, samples from the southern mountain areas; C, samples from the western areas; and D, samples from the Emei high platform areas. Symbols: triangle, shallow groundwater; circle, deep groundwater.

A rapid increase in the major ion concentration along the flow path was found in shallow groundwater (Fig 3), and the shallow groundwater from the central discharge areas contained the highest total dissolved solids (TDS) content of 17,452 mg/L, with Na^+^ as the dominant cation and SO_4_^2-^ and/or Cl^-^ as the dominant anion(s) (Table 1). The shallow groundwater from the northeastern piedmont plains (area A′) was generally classified as Na-HCO_3_/Na-Ca-HCO_3_ water, and that from the southern piedmont plains (area B′) was classified as Ca-HCO_3_ water, with a low content of TDS and low ion concentrations (Fig 4). Shallow groundwater from western areas (area C′) was of the Na-HCO_3_/Na-Ca-HCO_3_ and Na-/SO_4_ types and transitioned to Na-SO_4_ or Na-Ca-SO_4_ water in the flow-through areas. In the discharge areas, groundwater was typically of the Na-/SO_4_/Cl/SO_4_-HCO_3_ type in both shallow and deep aquifers.

The deep groundwater was alkaline, with a pH range of 7.3–8.6. Most of the deep groundwater samples consisted of freshwater with a TDS concentration below 1000 mg/L, belonging to the Na-HCO_3_ (Fig 4, subgroups A, C and D), Ca-HCO_3_ (subgroups A and C) or Ca-HCO_3_-SO_4_ (subgroup B) types in the recharge areas. Some deep groundwater samples (subgroup A) collected from the northeastern areas consisted of Ca-SO_4_/Na-Ca-SO_4_ water, which transitioned to Na-Ca-SO_4_ and Na-HCO_3_ water in the flow-through areas. A gradual increase in the ion concentrations was observed in deep groundwater along the flow path (Fig 3). The sources of solutes in the deep water are aquifer mineral dissolution, saline lake water intrusion and shallow groundwater leakage [37], [38], [44].

More than 70% of the shallow groundwater contained NO_3_^-^ in excess of 10mg/L. One groundwater sample (YH31) contained NO3- in excess of the WHO standard for drinking water. (50 mg/L). Because of the lack of natural nitrate in most geologic formations, NO_3_^-^ concentrations > 5 mg/L are generally indicative of water contamination by animal waste, fertilizer and/or effluent [45], [46]. The highest NO_3_^-^ content of 67.9 mg/L was observed in shallow groundwater, implying an unambiguous anthropogenic source of contaminants in this area. High NO_3_ concentrations were also observed in some deep groundwater samples, which indicates an exogenous input of contaminants from irrigation water and/or polluted shallow groundwater.

Stable isotopes δD and δ^18^O were analyzed in selected shallow groundwater, deep groundwater and surface water samples from the Yuncheng Basin. Table 1 summarizes the isotopic compositions (δ^18^O and δD) of selected water samples collected from the Yuncheng Basin during the study period. The surface water samples were more enriched in deuterium and ^18^O than the shallow and deep groundwater samples, suggesting that the surface water and shallow groundwater are systematically subjected to further fractionation by evaporation after rainfall episodes.

### Major geochemical processes

**Fig 5:**
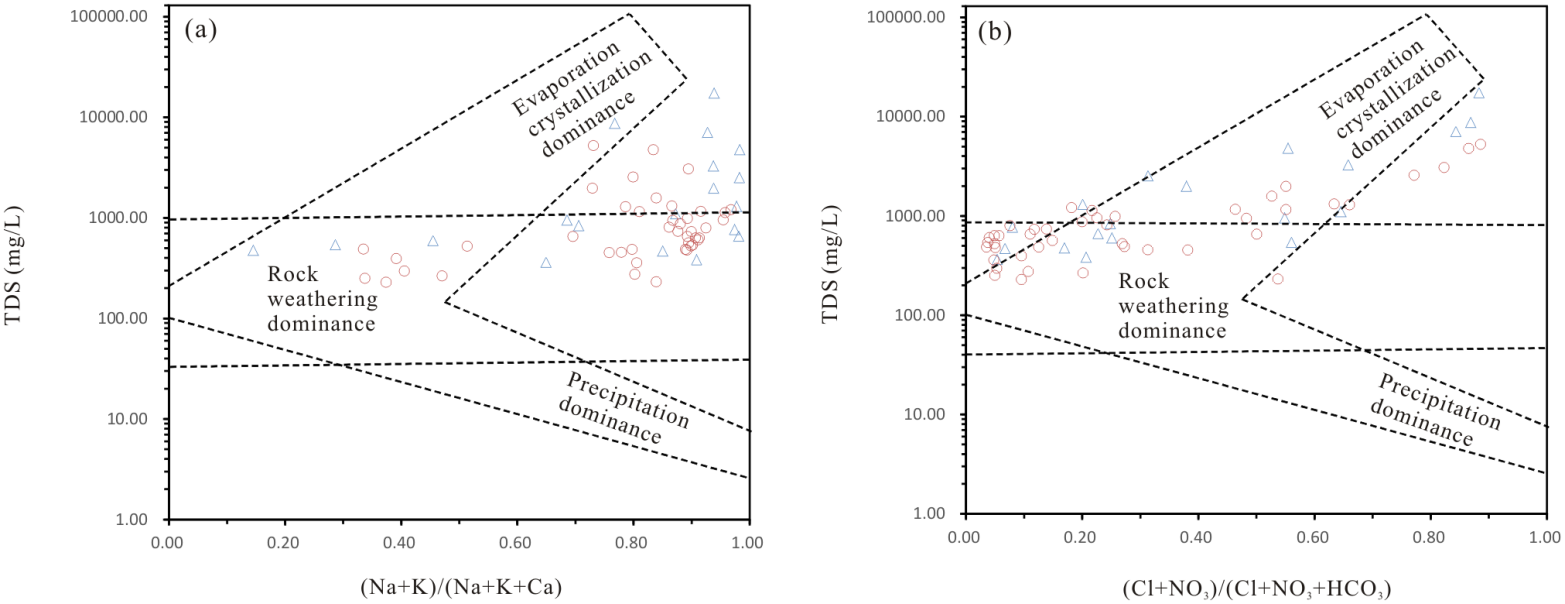
(a) Plots of TDS versus Na/(Na+Ca) and (b) TDS versus Cl/(Cl+HCO_3_) illustrating the dominant processes that control the groundwater chemistry in the study area. Triangle, shallow groundwater; circle, deep groundwater.

The mechanisms controlling groundwater chemistry and the functional sources of dissolved ions can be assessed by Gibbs diagrams [47], by plotting the TDS content versus the ratio of Cl/(Cl + HCO_3_) for anions and of (Na+K)/(Na+K+Ca) for cations. Gibbs diagrams illustrate the natural mechanisms controlling groundwater chemistry, including rock weathering dominance and evaporation and precipitation dominance. Based on the Gibbs diagram (Fig 5), most deep groundwater samples were influenced by rock weathering, with a TDS content below 1000 mg/L. Most shallow groundwater samples and some deep groundwater samples were evaporation dominant, indicating the important role of evaporation and/or the dissolution of evaporites, such as halite (NaCl), gypsum (CaSO_4_ ∙ 2H_2_O) and mirabilite (Na_2_SO_4_) [37], [48], in shallow groundwater chemistry. A few of the shallow groundwater samples, collected mainly from recharge and runoff areas, were rock weathering dominant, illustrating the significant impact of water-rock interactions on the chemistry of this groundwater. No samples were classified as precipitation dominant, suggesting a limited input of solutes from the atmosphere.

**Fig 6:**
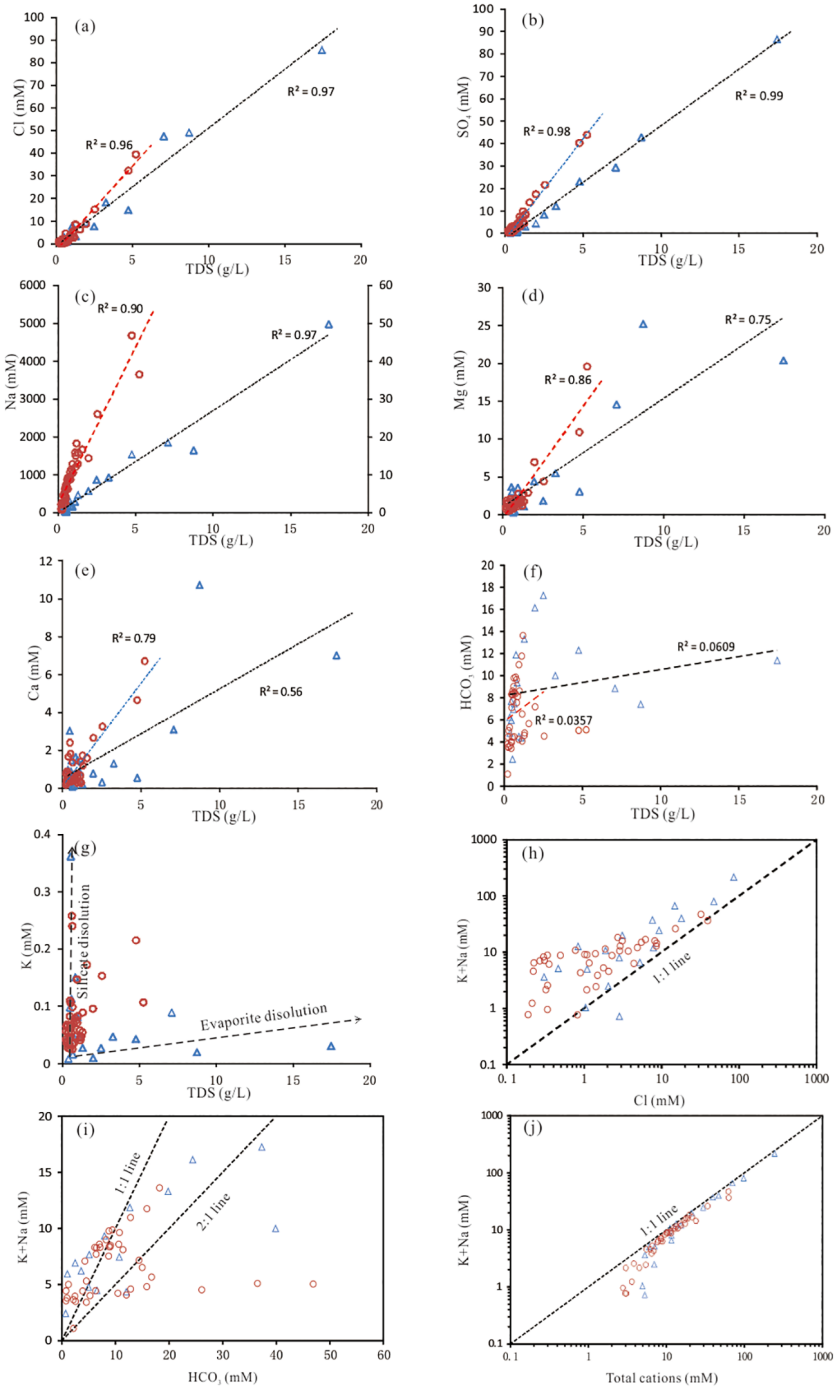
Cross-plots of the TDS and major ions in groundwater. Triangle, shallow groundwater; circle, deep groundwater; TSG, trend line for shallow groundwater; TDG, trend line for deep groundwater.

Cross-plots provide further insight into the possible ion sources in the groundwater of the Yuncheng Basin and are presented in Fig 6. Strong correlation existed between the major cations (Cl, SO_4_, Na, Ca and Mg) and TDS with coefficients (R^2^) of 0.56–0.99 (Fig 6a–6d), which is a clear indication of the contribution of these ions to the overall mineralization degree. A significant positive correlation was observed in the cross-plot of TDS versus HCO_3_ (Fig 6f), which may originate from carbonate or silicate dissolution. Some groundwater samples with high TDS contents (TDS > 3 g) did not show notable increases in the HCO_3_ concentration, which may be highly affected by evaporation or evaporite dissolution. In the cross-plot of TDS versus K (Fig 6g), the shallow groundwater samples were separated into two subgroups: 1) increased K concentration with a low TDS content, indicating silicate weathering, and 2) low K concentration (< 0.1 mM) with an increased TDS content, which can be explained by evaporation or evaporite dissolution. The deep groundwater samples fell into the area between the silicate weathering line and the evaporite dissolution line (Fig 6g), suggesting a combined effect of the dissolution of these minerals.

The significant impact of silicate weathering on groundwater chemistry was also indicated by the bivariate plots of HCO_3_ versus Na + K and Na + K versus total cations (Fig 6h and 6i). Most of the groundwater samples fell over the 1:2 line on the bivariate plot of HCO_3_ versus Na + K. Falling below the 1:1 line on the bivariate plot of Na + K versus total cations suggests that silicate weathering was a dominant process of introducing major ions in the groundwater (Fig 6i and 6j). In the plot of Na + K versus Cl (Fig 6h), most of the data clusters around the 1:1 line, illustrating the strong influence of the dissolution of evaporites, such as halite, on the groundwater chemistry. Hence, the ionic input into the groundwater system was most likely a consequence of geologic sources: natural water-rock interactions in sediment aquifers, especially the weathering dissolution of minerals in aquifers.

**Fig 7:**
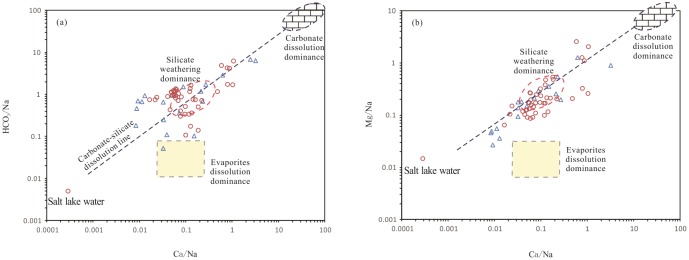
Molar ratio bivariate plots of (a) Na-normalized Ca and HCO_3_ and (b) Na-normalized Ca and Mg. The dashed circles represent the ranges of approximate compositions of the silicate and carbonate end members, and a dashed box represents the evaporite end member. Most deep groundwater samples fell in the area of silicate end member area, indicating that the majority of ions originated from silicate mineral weathering. Triangle, shallow groundwater; circle, deep groundwater; solid red dot, Salt Lake saline water.

To identify the relative contributions of the major weathering/dissolution mechanisms (silicate, carbonate, and evaporite) to the ion concentrations in groundwater, the Na-normalized molar ratio model (Ca/Mg/HCO_3_) suggested by Gaillardet et al. [49] was employed (Fig 7). In this model, waters draining carbonates (carbonate end member) in Ca- and Mg-dominated reservoirs have Ca/Na ratios close to 50, Mg/Na ratios close to 10, and HCO_3_/Na ratios close to 120; waters draining silicates (silicate end member) have lower Na normalized ratios, Ca/Na ratios of 0.35±0.15, Mg/Na ratios of 0.24±0.12, and HCO_3_/Na ratios of 2±1; and the evaporite end member (waters draining evaporites), which is difficult to constrain due to the diversity of salt rocks, is deplete in HCO_3_ ions relative to Ca and Mg ions, resulting in a downward deviation of their representative points from the silicate-carbonate line (Fig 7) [49].

The bivariate mixing plots (Fig 7) of Na-normalized HCO_3_ versus Ca and Na-normalized Mg versus Ca show that most of the deep well groundwater samples and some of the shallow groundwater samples were within or very close to the global average silicate weathering domain, which indicates a significant contribution of silicate mineral weathering dissolution to the groundwater chemistry of these samples. However, some of the shallow groundwater and deep groundwater samples plotted in the transitional area between the carbonate end member and silicate weathering zone, illustrating a combined effect of carbonate and silicate dissolution. Resulting from the dissolution of evaporites, which are deplete in HCO_3_ ions, a downward deviation of some shallow and deep groundwater samples from the silicate-carbonate mixing line was observed in the bivariate mixing plots (Fig 7), which indicates a significant contribution of evaporite dissolution to the groundwater chemistry of these samples. This interpretation is supported by the findings of our field investigation and other studies, in which soluble evaporites, such as halite, bloedite, and gypsum, are widely distributed in Middle Pleistocene sediments in the Yuncheng Basin [37], [48].

**Fig 8:**
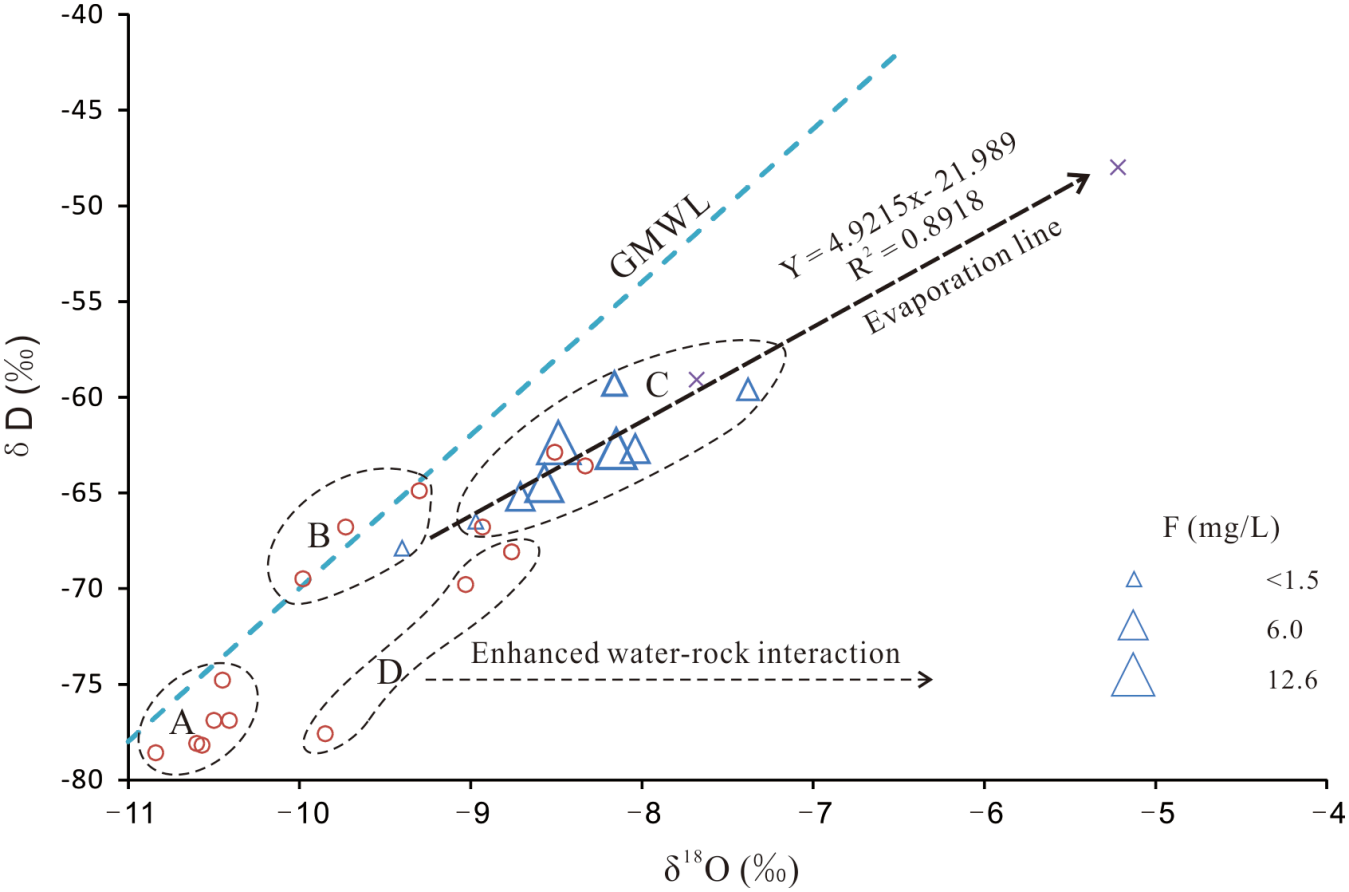
Scatterplot of δ^18^O versus δD for selected water samples. Triangle, shallow groundwater; circle, deep groundwater; X-shape, surface water; Subgroup A, groundwater recharged by high-altitude precipitation in the mountain areas; Subgroup B, groundwater recharged by rainwater in a lower altitude area; Subgroup C, waters subjected to evaporation; Subgroup D, groundwater affected by extensive water-rock interactions.

Evaporation, as suggested by the Gibbs diagram (Fig 5), is another geochemical process that potentially affected the groundwater chemistry in the area. The effect of evaporation on groundwater chemistry is confirmed by the significant deviation of the δ^18^O and δD values of most shallow groundwater samples about the global meteoric water line (GMWL) (Fig 8). The groundwater samples that plotted along the GMWL were separated into several subgroups according to their positions. Subgroup A, located in the left lower corner of the Fig with more negative δD and δ^18^O values, is representative of groundwater recharged by high-altitude precipitation in the southeastern and northern mountain areas (Fig 1). Subgroup B, located above the GMWL, was composed of groundwater samples recharged by rainwater in a lower altitude area. Subgroup C, consisting of surface water, shallow groundwater and several deep groundwater samples, deviated to the right of the GMWL, which is indicative of water typically subjected to evaporation, being enriched in ^18^O and deuterium and resulting in a lower slope (Y = 4.92X − 21.99). It is impossible for deep groundwater to undergo evaporation due to the large burial depth. The presence of some deep groundwater samples in this group suggests a possible recharge from surface water or shallow groundwater that had been affected by evaporation. Subgroup D, lying parallel to the GMWL, was composed of several deep groundwater samples and is indicative of the effect of extensive water-rock interactions (horizontal arrow).

### Fluoride contamination of groundwater

#### Occurrence of fluoride

The fluoride concentrations in precipitation, surface water and groundwater were analyzed in this case study. Precipitation had a low fluoride content due to the absence of mineral inputs (Table 1). Generally, seawater spray and atmospheric pollutants are the major sources of fluorine in precipitation. Atmospheric pollution is likely the source of fluoride in rainwater in the Yuncheng Basin because it is an inland catchment located far from the ocean.

The surface water fluoride concentration ranged from 0.32 to 15.36 mg/L, with an average of 5.01 mg/L. The fluoride concentration was low in the surface water samples collected from the mountain areas and piedmont plain areas. The surface water samples with a high fluoride concentration were all collected from areas with heavy human activity, such as saline lakes and reservoirs. This suggests that human activities may be partly responsible for the increase in the fluoride concentration in natural water systems; similar conclusions were drawn by Machender et al. [50], Pathak [51], and Ravichandran et al. [52].

**Fig 9:**
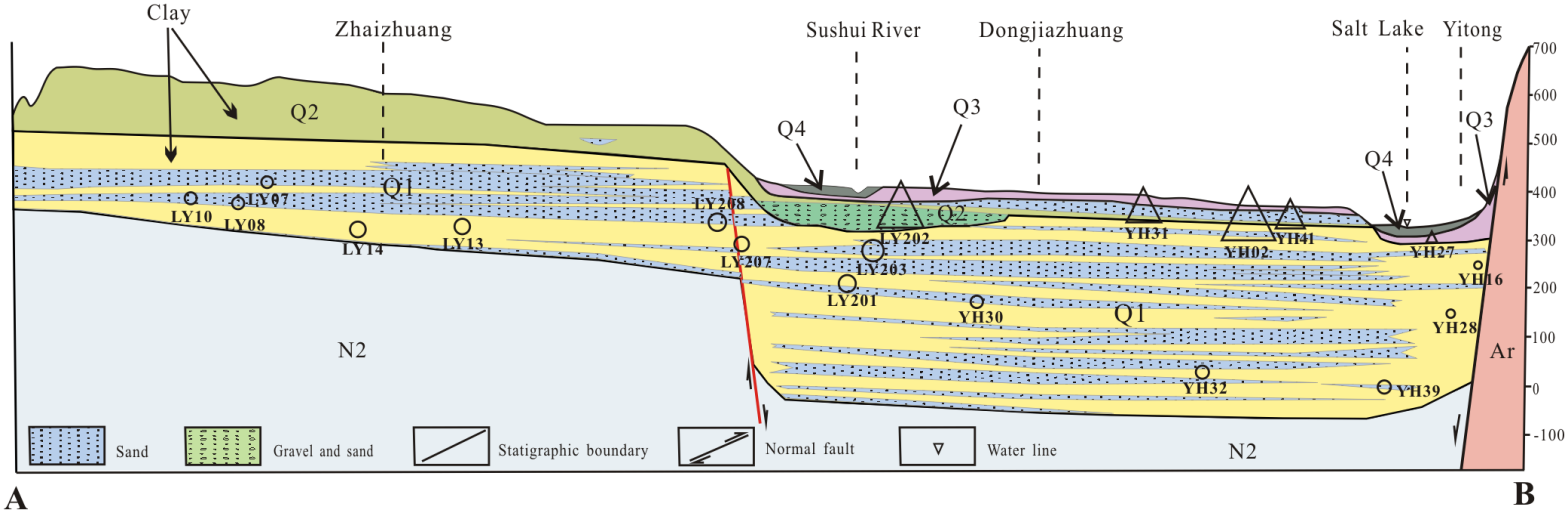
Distribution of fluoride in groundwater with depth along profile AB in the Yuncheng Basin. Areas of circles or triangles represent the concentration of fluoride in groundwater, as in Fig 1. triangle, shallow groundwater; circle, deep groundwater.

Groundwater in the Yuncheng Basin suffers from heavy fluoride pollution (Figs 1 and 9). Approximately 60% of the shallow wells had fluoride contents above the WHO provisional drinking water limit of 1.5 mg/L. The concentration of fluoride in groundwater is not uniform in the study area, varying between 0.53 mg/L and 12.65 mg/L, with an arithmetic mean of 4.18 mg/L (Table 1). The lowest fluoride concentration was detected in shallow groundwater from the piedmont plain area, where the groundwater was mainly recharged by precipitation and fissure water (Figs 1 and 9). A high fluoride content was observed in shallow groundwater from the central basin area. This groundwater normally has a low to moderate calcium content and medium to high TDS content.

The deep groundwater samples were characterized by low to medium F^−^ concentrations that ranged from 0.1 mg/L to 3.15 mg/L (Table 1). Approximately thirty percent of the deep wells had fluoride contents that exceeded 1.5 mg/L (limit recommended by the WHO). The wide distribution of the fluoride concentration in deep and shallow groundwater suggests that the fluoride pollution in this area is non-point source pollution.

The spatial variation of the fluoride concentration is presented in Figs 1 and 9. Physiographically, the high-fluoride groundwater is more concentrated in the central areas than in the margins along the flow path of groundwater movement.

**Fig 10:**
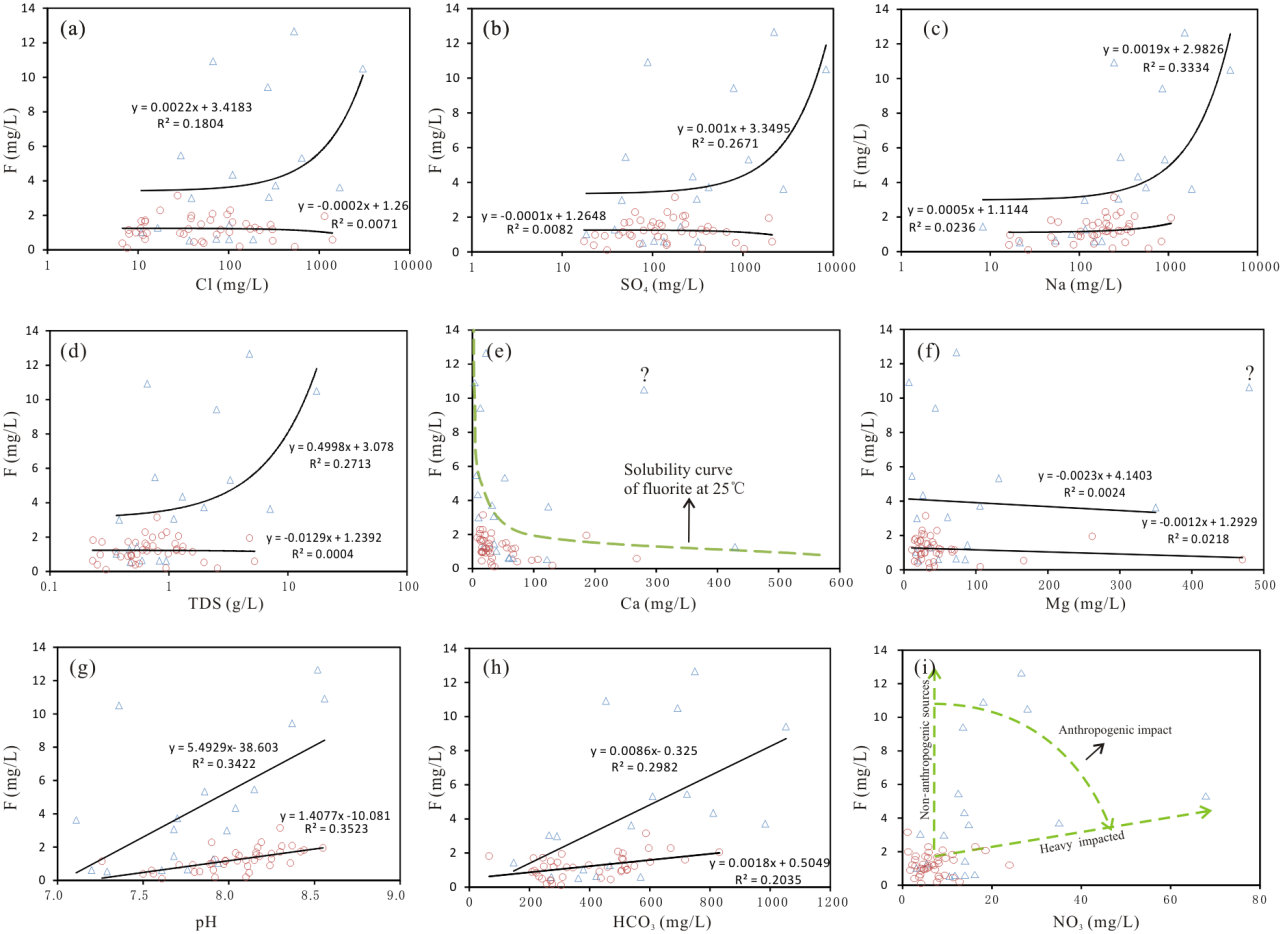
Scatterplot of the fluoride concentration versus the concentrations of major ions, the TDS content and the pH of groundwater in the Yuncheng Basin. Δ, shallow groundwater; ○, deep groundwater.

The hydrochemistry of fluoride-polluted groundwater (F > 1.5 mg/L) is shown in Fig 10. The fluoride-rich groundwater was typically alkaline (7.0 < pH < 9.0) with a medium to high HCO_3_ content (mean of 539.6 mg/L in shallow groundwater and 399.2 mg/L in deep groundwater), medium to high SO_4_ and Cl contents, high Na content (mean of 752.8 mg/L in shallow water and 230.2 mg/L in deep groundwater), and low to medium Ca content. These results indicate that alkaline conditions, competitive adsorption, cation exchange, salt effects and the removal of Ca by precipitation are vital factors controlling the enrichment of fluoride in groundwater in the study area.

#### Geochemical processes controlling groundwater fluoride contamination

Sources of fluoride. The typical fluorine-rich rocks are syenites, granites, quartz monzonites, granodiorites, felsic and biotite gneisses, and alkaline volcanics, with fluorite (CaF_2_), fluoroapatite (Ca_5_(PO_4_)_3_F), micas, amphiboles, cryolite (Na_3_AlF_6_), villiaumite (NaF) and topaz (Al_2_(SiO_4_)F_2_) as the major fluoride-bearing minerals [53–61]. The fluoride concentration is positively correlated with the pH and the TDS, Cl, SO_4_, HCO_3_ and Na concentrations (Fig 9) in the groundwater samples, indicating a natural mineral weathering dissolution source of fluoride in groundwater.

**Fig 11:**
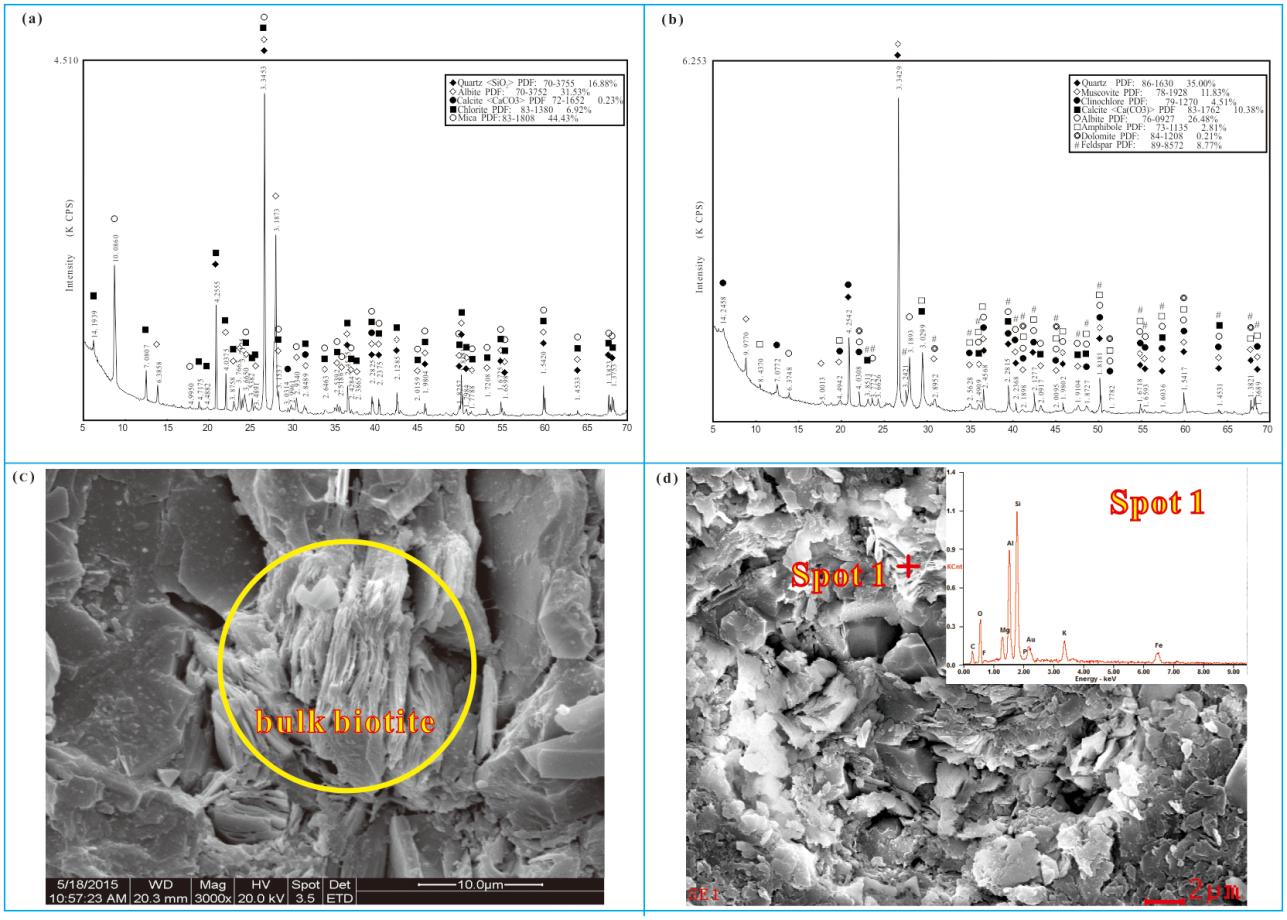
SEM and XRD analyses of rock/sediment samples from the Yuncheng Basin. **(a) XRD pattern of a rock sample, (b) XRD pattern of a sediment sample, (c) SEM pattern of a rock sample, (d) SEM pattern of a sediment sample and chemical components of point one in** Fig 8d**.** Biotite/micas were identified as major fluoride-bearing minerals in both rock and sediment samples.

In the Yuncheng Basin, the major fluoride-bearing minerals were identified to be micas and amphibole in Quaternary sediments (Table 2) and biotite in metamorphic rock (Fig 11). The total fluorine content ranged between 220–1,300 mg/kg in sediments and rocks (Table 3). Biotite schist had the highest fluoride content, with biotite as the major fluoride-bearing mineral, which is the major weathering mineral source of fluorine to the subsurface environment. The clay and silt sediments also had high fluoride contents of 1,118 and 1,106 mg/kg, respectively (Table 3). Due to the high adsorptivity of fluoride on clay minerals, the exchangeable fluoride and the fluoride in the mineral matrix may account for a significant portion of the total fluorine in these materials. The adsorbed fluoride could be easily released from the clay/silt sediments in certain environments, such as alkaline conditions or soda-rich water. The release of fluoride from sediment may be a major source of fluoride in groundwater [62], [63].

**Table 2:** Summary of the major minerals in sediments (n = 50) from the Yuncheng Basin.

Minerals (weight percent)	Max.	Min.	Mean	SD.
**Quartz**	68.25	19.71	40.02	9.40
**Chlorite**	14.14	0.26	4.12	2.95
**Mica**	31.52	0.86	11.71	6.69
**Calcite**	44.38	0.24	8.79	5.83
**Albite**	41.09	1.58	23.10	8.26
**K-feldspar**	43.26	0.17	8.96	7.61
**Amphibole**	5.50	0.45	2.15	0.95
**Dolomite**	9.60	0.09	1.50	2.10

**Table 3:** Fluoride content (mg/kg) in rock/sediment samples (n = 3) from the Yuncheng Basin.

	Rock/sediments	Average content of F (mg/kg)	SD
**Rock type**	**Biotite schist**	1300	200.0
	**Granite porphyry**	803.0	69.35
	**Granodiorite porphyry**	553.0	29.57
	**Dolomite**	260.0	22.52
	**Biotite granite**	437.0	84.25
	**Basalt**	520.0	85.06
	**Calcite**	220.0	50.01
**Sediment type**	**Clay**	1118	112.0
	**Silt**	1106	68.15
	**Fine sand**	535.0	71.63
	**Medium sand**	426.7	36.42

Processes controlling fluoride contamination. In general, fluoride is preferentially adsorbed onto sediment mineral surfaces under neutral conditions. Abundant research indicates that pH is one of the major factors that governs the liberation and mobility of fluoride into groundwater. A significant positive correlation was observed between the pH and fluoride concentration in groundwater (Fig 10g) in the Yuncheng Basin. The mechanisms of this pH control may include the following: (1) High pH could affect the ionic charge of F^-^ and the properties of solid surfaces and further promote the desorption/adsorption of anions [64–66]. (2) Considerable OH^-^ in groundwater could cause Ca^2+^, Fe^3+^ and Al^3+^ to precipitate, preventing F^-^ from complexing with cations, which results in a high release of F^-^ in groundwater. (3) Under alkaline conditions, OH^-^ could exchange with F^-^ adsorbed on clay minerals, humus and soil colloids [67–70]. Thus, the interaction between pH and fluoride partly explains the distribution of fluoride in groundwater.

Competitive adsorption by other anions, such as HCO_3_, could cause the desorption of fluoride from mineral/organic matter surfaces within the groundwater system [71], [72]. A decrease in fluoride adsorption of 32.2% with an increase in the bicarbonate concentration of 300 mg/L was reported by Alagumuthu et al. [73], reflecting the great ability of bicarbonate to compete with fluoride for active sites. In this case study, a slight positive correlation was observed between the HCO_3_ and fluoride concentrations in groundwater, and the groundwater samples highly contaminated with fluoride ([F] > 4.0 mg/L) all had high bicarbonate concentrations between 454.6 and 1,053 mg/L, illustrating the intense competitive adsorption between fluoride and HCO_3_ at the active sites (Fig 10h).

Evaporation/evapotranspiration is another important process that increases the fluoride concentration in groundwater in arid and semi-arid regions [18]. A significant linear relationship between the fluoride and Cl (a conservative element) concentrations (Fig 10a) suggests that evaporation is one of the major processes occurring in the groundwater of the Yuncheng Basin. The effect of evaporation and condensation on the fluoride contamination in shallow groundwater was confirmed by the δD and δ^18^O scatterplots (Fig 8), where a right deviation of δD and δ^18^O from the GMWL and an increased fluoride concentration were observed. Most of the shallow groundwater samples fell on the evaporation line, illustrating the significant effect of evaporation on groundwater chemistry. Elevated fluoride concentrations were detected in these shallow groundwater samples in subgroup C (samples greatly affected by evaporation), illustrating that evaporation could partly contribute to the contamination of fluoride in these groundwaters. The mechanisms by which evaporation increases the fluoride concentration may include the following: 1) Evaporation could directly remove water from shallow aquifers [74], [75], elevating the fluoride concentration in groundwater [16]. 2) Evaporation could increase ion concentrations, leading to the precipitation of some major minerals [33], [76], [77], e.g., calcite and dolomite, reducing the Ca concentration and favoring the dissolution of fluorite and the enrichment of fluoride in groundwater. Deep groundwater is not affected by evaporation due to the great burial depth; therefore, the appearance of three deep groundwater samples in subgroup D group is perhaps due to the leakage of shallow groundwater, irrigation return water and/or surface water that had been subjected to evaporation.

**Fig 12:**
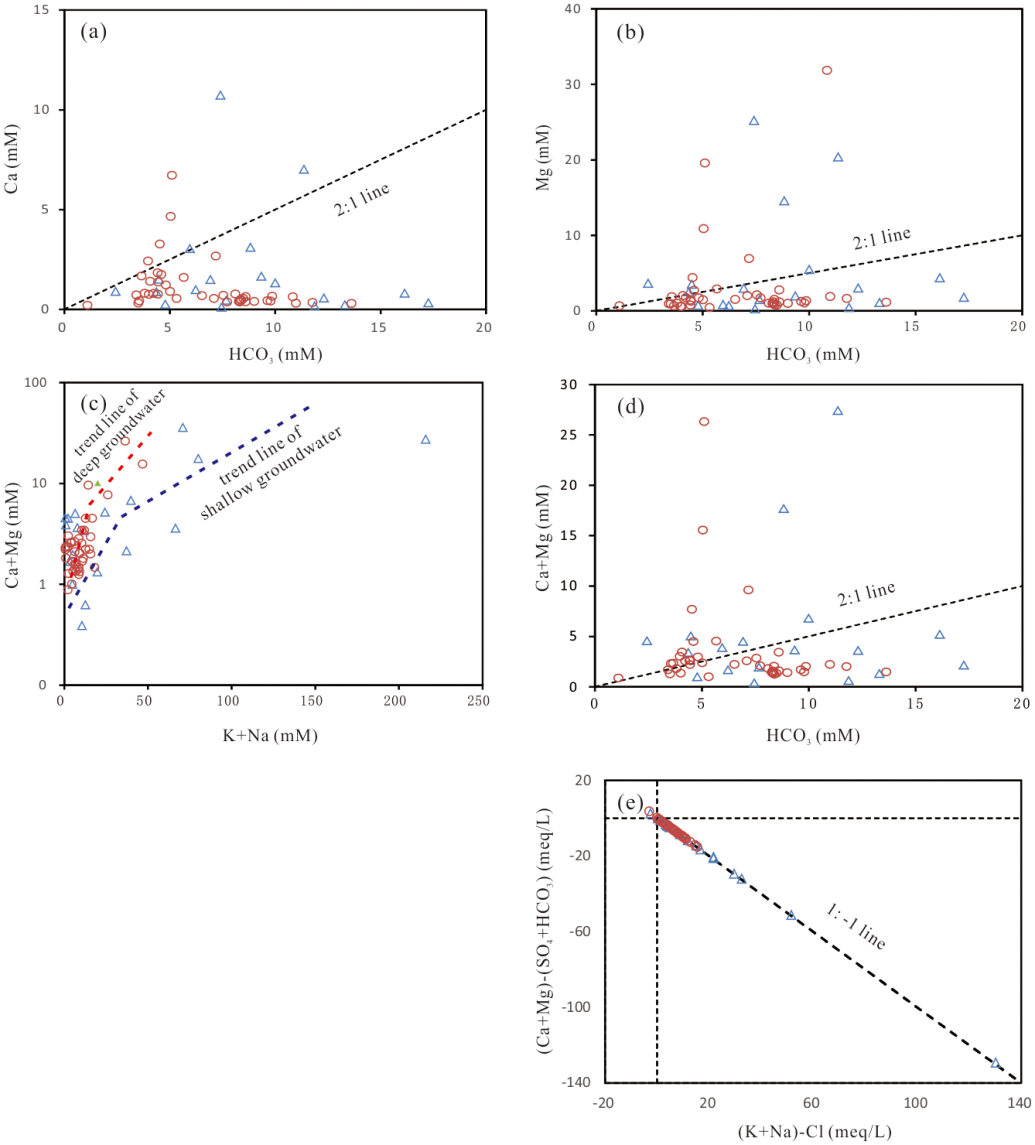
Bivariate plots of the major ions in groundwater. **(a) Ca + Mg versus HCO**_**3**_**; (b) Ca versus HCO**_**3**_**; (c) Mg versus HCO**_**3**_**; (d) Ca + Mg versus K+Na; (e) (Ca+Mg)-(SO**_**4**_**+HCO**_**3**_**) versus K+Na-Cl.** triangle, shallow groundwater; circle, deep groundwater.

In addition to evaporation in shallow groundwater, cation exchange, ion effects and salt effects are important geochemical factors that cause fluoride pollution by reducing the Ca content in groundwater. Cation exchange occurs widely in sedimentary aquifers and involves of the replacement of the bivalent cations Ca and Mg in the aquifer matrix with the monovalent cations Na and K in groundwater [78]. Bivariate plots of Ca, Mg, and Ca+Mg versus HCO_3_; Ca+Mg versus Na+K; and Ca+Mg-SO_4_-Cl versus Na+K-Cl (Fig 12a–12d) were employed to show the influence of cation exchange in which the monovalent cations Na and K in groundwater replaced the bivalent cations Ca and Mg in the aquifer matrix. Most of the groundwater samples fell over the 1:2 line on the bivariate plot of HCO_3_ versus Ca, Mg and Ca+Mg, suggesting cation exchange as a dominant process in groundwater chemistry, in addition to silicate weathering. The bivariate plot in Fig 12e confirmed the influence of cation exchange—the trend line with slope of ca. -1 indicates that cation exchange probably has a strong influence on the groundwater chemistry of the study area. In this way, the concentration or molar ratio of Ca is apparently reduced in groundwater, consequently promoting the dissolution of fluoride-bearing minerals in aquifers and increasing the fluoride concentration in groundwater. A rough estimation of the contribution of cation exchange to fluoride contamination was obtained by calculating the increase in the fluoride concentration with the increase in the Na/Ca molar ratio in simulated solutions (calculation performed with PHREEQC V2.0.1.8). Two groundwater solutions, one Na-HCO_3_ water with a Na/Ca molar ratio of 1.17 and one Ca-HCO_3_ water with a Na/Ca molar ratio of 0.24, were used as the initial solutions in the simulations. The simulation results show that an increase in the Na/Ca molar ratio from 0.24 to 9.06 caused an increase of 2.7 mg/L fluoride in solution one, and an increase in the Na/Ca molar ratio from 2.0 to 8.99 caused an increase of 2.82 mg/L fluoride in solution two by equilibration with fluorite.

**Fig 13:**
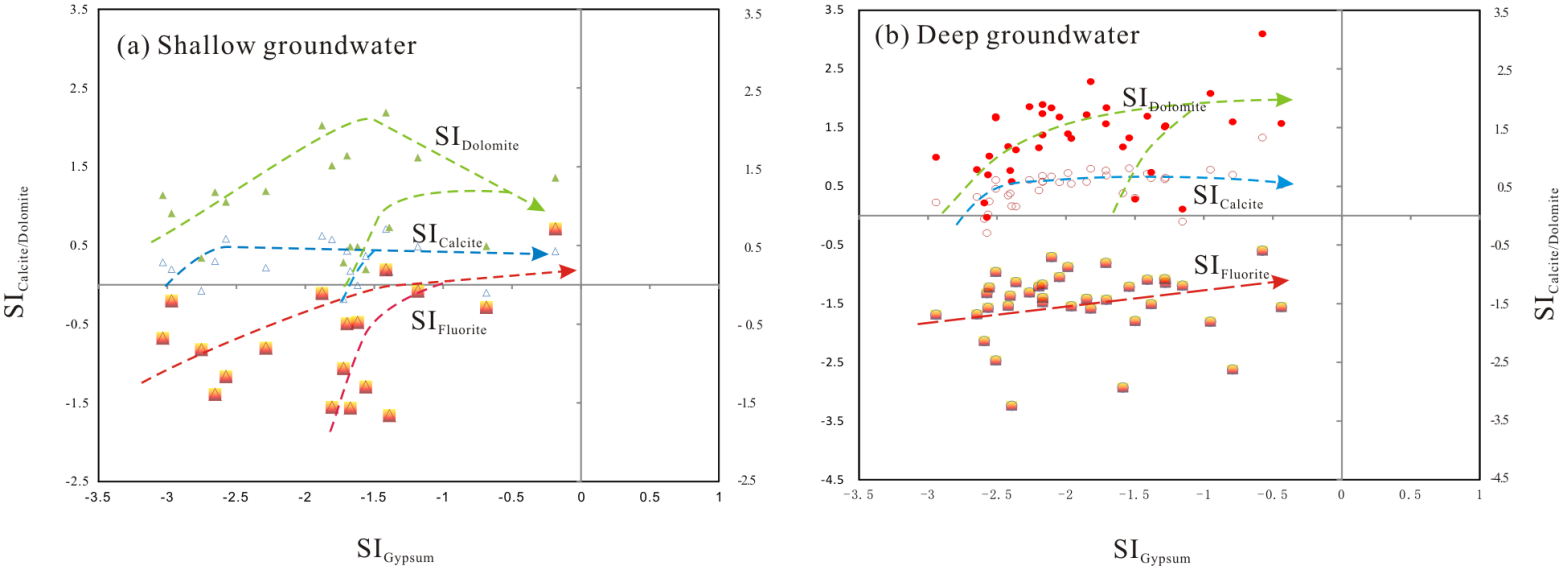
A bivariate plots of SI_Dolomite_/SI_Calcite_ versus SI_Gypsum_ in shallow groundwater (a) and deep groundwater (b). Meaning of symbols: the green solid triangle, SI_Dolomite_ of shallow groundwater; the blue hollow triangle, SI_Calcite_ of shallow groundwater; the big solid red triangle, SI_Fluorite_ of shallow groundwater; the small solid red circle, SI_Dolomite_ of deep groundwater; the big hollow red circle, SI_Calcite_ of deep groundwater; the big solid red circle, SI_Fluorite_ of deep groundwater.

Ion effects were induced by the dissolution of deposited evaporites, especially gypsum, in the aquifer sediments in the study area. The dissolution of gypsum introduces additional calcium into the groundwater system and causes the precipitation of calcite and dolomite from the groundwater [79], [80], by which groundwater maintains a low level of calcium and a constant dissolution capacity for fluorite. The saturation of groundwater with respect to gypsum, calcite and dolomite is illustrated in Fig 13. The mineral phase of gypsum is undersaturated in the groundwater, indicating that the mineral phase will tend to dissolve. Calcite and dolomite are distributed around the saturation/equilibrium or oversaturation areas in the scatterplot. The mineral phases that are oversaturated (SI ≥ 0.1) will precipitate out of solution. However, an increase in the SI of calcite, dolomite and gypsum in Fig 13 implies dissolution of the minerals. The dissolution of gypsum and dolomite and the subsequent exchange between cations lead to the precipitation of calcite, as shown by the constant SI_Calcite_ value with the increase in SI_Dolomite_ and SI_Gypsum_ (Fig 13). The removal of Ca from groundwater by calcite precipitation occurs within the groundwater system and increases the HCO_3_ concentration via further dissolution of dolomite. The constant increases in SI_Fluorite_ of groundwater indicate the further dissolution of fluorite and consequent increase in the fluoride concentration in the groundwater. This elevated fluoride concentration is attributed to the ion effects in groundwater.

Salt effects promote the dissolution of most fluoride-bearing minerals in aquifers by reducing the activity of fluoride in groundwater via ion complexation. A positive correlation between F and Cl, SO_4_, Na and TDS (Fig 10a–10d) suggests that salt effects could be one of the main factors controlling the presence of fluoride species in the groundwater of the Yuncheng Basin. The source of salt may be either evaporite (such as halite, gypsum, mirabilite and bloedite) dissolution or saline lake water intrusion, as previously reported by Gao et al. [37]. A rough calculation of the contents of the fluoride species in the groundwater shows that complex fluoride accounts for up to 6.02–19.38% of the total fluorine in saline groundwater but only 1.33–8.16% in fresh groundwater (Table 4). MgF^+^ was the most abundant complex fluoride species in both fresh and saline groundwater, and the highest percentage of MgF^+^ was found in saline groundwater (15.92%). NaF^0^ was the second most abundant fluoride species (up to 4.31%) in saline groundwater, while CaF^+^ was the second most abundant species in fresh groundwater, indicating an increased formation of Na^+^-F^-^ complexes in the form of NaF^0^ at the extremely high sodium concentrations in saline groundwater. The percentages of HF^0^ and BF(OH)_3_^-^ were higher in the saline groundwater than in the fresh groundwater. Therefore, increases in the ion concentrations in saline groundwater favor the formation of complex fluoride, reduce the activity of aqueous fluoride, and promote the further dissolution of fluoride-bearing minerals or the desorption of fluoride from mineral/organic matter surfaces.

**Table 4:** Percentages of fluoride species in selected fresh and saline groundwater samples (calculated by PHREEQC).

Water type	Fresh groundwater												Saline groundwater							
Sample ID	YJ-01		YJ-02		YJ-03		YJ-05		YJ-08		LY2-11		YJ-15		LY2-10		LY2-12		YH02	
Species	Molality	Percentage	Molality	Percentage	Molality	Percentage	Molality	Percentage	Molality	Percentage	Molality	Percentage	Molality	Percentage	Molality	Percentage	Molality	Percentage	Molality	Percentage
**Total F**	5.37E-06		1.69E-05		9.47E-06		3.40E-05		2.05E-05		5.75E-04		5.63E-04		1.92E-04		1.03E-04		6.69E-04	
**F**^**-**^	5.07E-06	94.45	1.66E-05	98.17	8.70E-06	91.85	3.15E-05	92.53	1.93E-05	94.34	5.67E-04	98.66	4.54E-04	80.55	1.57E-04	81.72	8.57E-05	83.17	6.29E-04	94.01
**MgF**^**+**^	2.78E-07	5.18	8.01E-08	0.47	7.31E-07	7.72	2.41E-06	7.10	1.08E-06	5.25	4.95E-06	0.86	8.12E-05	14.43	3.06E-05	15.92	1.51E-05	14.70	2.58E-05	3.86
**CaF**^**+**^	2.17E-08	0.40	2.18E-07	1.29	3.44E-08	0.36	9.11E-08	0.27	7.24E-08	0.35	2.72E-06	0.47	3.51E-06	0.62	8.09E-07	0.42	8.33E-07	0.81	5.95E-07	0.09
**NaF**	2.78E-09	0.05	5.89E-09	0.03	6.50E-09	0.07	1.18E-08	0.03	6.52E-09	0.03	2.14E-07	0.04	2.43E-05	4.31	3.87E-06	2.02	1.34E-06	1.30	1.38E-05	2.06
**HF**	1.51E-10	0.0028	2.19E-10	0.0013	4.71E-10	0.0050	1.26E-09	0.0037	7.50E-10	0.0037	1.83E-09	0.0003	1.65E-08	0.0029	1.13E-08	0.0060	2.51E-10	0.0002	1.87E-09	0.0003
**BF(OH)**_**3**_^**-**^	9.91E-12	0.0002	5.27E-11	0.0003	2.25E-11	0.0002	1.32E-10	0.0004	2.05E-11	0.0001	3.56E-08	0.0062	7.35E-08	0.0131	1.24E-08	0.0065	1.88E-09	0.0018	7.18E-08	0.0107
**F complexed**	3.03E-07	5.64	3.04E-07	1.33	7.73E-07	8.16	2.52E-06	7.41	1.16E-06	5.64	7.92E-06	1.38	1.09E-04	19.38	3.53E-05	18.37	1.73E-05	16.81	4.03E-05	6.02

Anthropogenic contamination. Several human activities may also result in substantial fluoride inputs to the subsurface aquatic environment [9], [81–83]. One of the significant sources of fluoride pollution is the use of domestic sewage, fertilizer and pesticides in agriculture in the Yuncheng Basin. Some shallow groundwater samples in Fig 10i are characterized by high NO_3_^-^ concentrations and a moderately high fluoride concentration. These samples were mainly collected in rural areas where a great deal of domestic sewage, agricultural fertilizer and pesticides had been discharged into the nearby ground or ditches, infiltrating the groundwater. Long-term irrigation with domestic sewage is a major source of manure for agricultural activities in the area, and nitrogenous fertilizer is another important source of nitrogen in local agriculture. Both of these practices could cause elevated NO_3_^-^ concentrations in the groundwater of this widely distributed agricultural areas [84], [85]. Therefore, anthropogenic pollution is responsible for the high NO_3_^-^ concentrations and elevated fluoride concentration in the shallow groundwater samples in Fig 10i. Table 5 shows the fluoride contents in the fertilizer and pesticides samples collected in the study area. The total fluorine content and the water-soluble fluoride content ranged between 3.19 and 1161 mg/kg and 0.57 and 51.87 mg/L, respectively, which reflect persistent groundwater fluoride pollution in the area.

**Table 5:** Fluoride content in selected pesticides/fertilizer/solid waste samples in the study area.

	ID	Fertilizer/pesticide/solid waste	pH	Water-soluble fluoride (mg/L)			Total fluorine (mg/kg)		
				concentration	mean	SD	concentration	mean	SD
**Pesticides**	NY01	DDT (dichlorodiphenyltrichloroethane)	6.1	21.91	-	-	21.91	34.23	12.62
	NY02	Acetamiprid	6.9	51.87			51.87		
	NY03	Beta cypermethrin	7.1	30.92			30.92		
	NY04	Avermectin	7.2	32.2			32.2		
**Fertilizers**	HF01	Foliar fertilizer	6.4	1.85	6.96	9.73	92.32	343.01	490.45
	HF02	Compound fertilizer	6.5	23.22			1161		
	HF03	Nitrogenous fertilizer A	6.7	0.62			31.11		
	HF04	Nitrogenous fertilizer B	6.2	14.84			741.9		
	HF05	Urea	7.5	0.57			28.53		
	HF07	Potassium ammonium nitrate	5.8	0.64			3.19		
**Solid waste**	GF01	Magnesium plant	10.25	1.93	2.46	1.88	543.18	694.81	178.32
	GF02	Paper mill	8.7	1.54			546.97		
	GF03	Steel mill	12.08	7.52			465.91		
	GF05	Cement plant	9.24	1.13			933.93		
	GF09	Paper mill	8.16	2.51			656.87		
	GF10	Glassworks	8.12	1.39			563.05		
	GF14	Pump industry	7.99	1.46			838.4		
	GF16	Chemical factory	7.91	1.46			933.18		
	GF17	Juice factory	7.81	1.25			465.56		
	GF18	Power plant	7.29	3.38			889.23		
	GF19	Industrial park for aluminum processing	7.75	4.47			715.36		
	GF20	Pesticide factory	7.56	1.46			786.11		

Many of the industries with fluoride pollution problems are also sources of groundwater fluoride pollution [86], [87]. Industries often produce fluoride-laden solid waste that may undergo open-air leaching before disposal. Aluminum and steel industries in the Yuncheng Basin produced solid waste with the highest water-soluble fluoride contents of up to 7.52 and 4.47 mg/L, respectively. Fluoride discharges from other industries were not negligible but were smaller than those from steel and aluminum operations (Table 5). The infiltration of wastewater/leachate with a high fluoride content is likely another anthropogenic source of groundwater fluoride contamination in the study area based on the results of our field investigation and laboratory results.

## Conclusions

The groundwater chemistry in the studied area showed wide variations in ion concentrations and hydrochemical facies, indicating complex hydrochemical conditions in the Yuncheng Basin. Sediment mineral weathering leaching and evaporite dissolution were the primary hydrochemical processes controlling the groundwater chemistry. Deep groundwater was contaminated by mixing with polluted surface water, irrigation return water and/or shallow groundwater.

Fluoride pollution also has important impacts on the groundwater chemistry in this area. The sources of fluoride in groundwater can include fluoride-bearing mineral dissolution, ion exchange, desorption from sorbent surfaces and anthropogenic contamination. The biotite/muscovite widely distributed in the sediment and base rock was determined to be the most abundant mineral fluoride source in the study area. Alkaline groundwater modified the properties of solid surfaces and promoted the desorption and exchange of fluoride with other anions. Massive anions, such as HCO_3_, could cause the desorption of fluoride from mineral/organic matter surfaces by competitive adsorption on the active sites within the groundwater system. Evaporation/evapotranspiration is critical for the concentration of fluoride in groundwater in this arid and semi-arid region. These processes can directly remove water from shallow aquifers; increase the fluoride concentration in groundwater; induce the precipitation of major minerals, e.g., calcite and dolomite; decrease the Ca concentration; and favor the dissolution of fluorite and the enrichment of fluoride in groundwater. Except for evaporation, ion effects induced by the dissolution of evaporites (gypsum) introduced additional calcium into the groundwater system and caused the precipitation of calcite/dolomite from the groundwater, which decreased the activity of calcium and maintained the dissolution capacity for fluorite in the groundwater. A rough estimation revealed that cation exchange may cause a significant increase of 2.7 mg/L fluoride when the Na/Ca molar ratio increases from 0.24 to 9.0. The promoting mechanisms of salt effects in fluoride-polluted water were the further dissolution of most fluoride-bearing minerals in aquifers by a reduction in the fluoride activity in groundwater via ion complexation. MgF^+^ and NaF^0^ are the most abundant complex fluoride species in saline groundwater and account for 6.0–19.0% of the total fluorine.

Human activities (domestic sewage, fertilizer and pesticide application in agriculture) and industries that cause fluoride pollution (aluminum and steel industries) may result in substantial fluoride inputs to the subsurface aquatic environment based on our field investigation and laboratory results.

## Supporting information

S1 Table Percentages of fluoride species in selected fresh and saline groundwater samples (calculated by PHREEQC). (DOCX)

S2 Table General characteristics of groundwater samples. (DOCX)
