# Supplementary material for: Geochemical processes controlling the groundwater chemistry and fluoride contamination in the Yuncheng Basin, China—An area with complex hydrogeochemical conditions
Source: PLoS One. 2018 Jul 26;13(7):e0199082. doi: 10.1371/journal.pone.0199082 (PMC6062146; doi:10.1371/journal.pone.0199082)
Supplement: S1 Table — (DOCX) [file pone.0199082.s001.docx]

S1 Table. Percentages of fluoride species in selected fresh and saline groundwater samples (calculated by PHREEQC)

| Water type | Fresh groundwater | | | | | | | | | | | |  | Saline groundwater | | | | | | | |
| --- | --- | --- | --- | --- | --- | --- | --- | --- | --- | --- | --- | --- | --- | --- | --- | --- | --- | --- | --- | --- | --- |
| Sample ID | YJ-01 | | YJ-02 |  | YJ-03 |  | YJ-05 |  | YJ-08 | | LY2-11 | |  | YJ-15 | | LY2-10 | | LY2-12 | | YH02 | |
| Species | Molality | Percentage | Molality | Percentage | Molality | Percentage | Molality | Percentage | Molality | Percentage | Molality | Percentage |  | Molality | Percentage | Molality | Percentage | Molality | Percentage | Molality | Percentage |
| Total F | 5.37E-06 |  | 1.69E-05 |  | 9.47E-06 |  | 3.40E-05 |  | 2.05E-05 |  | 5.75E-04 |  |  | 5.63E-04 |  | 1.92E-04 |  | 1.03E-04 |  | 6.69E-04 |  |
| F^-^ | 5.07E-06 | 94.45 | 1.66E-05 | 98.17 | 8.70E-06 | 91.85 | 3.15E-05 | 92.53 | 1.93E-05 | 94.34 | 5.67E-04 | 98.66 |  | 4.54E-04 | 80.55 | 1.57E-04 | 81.72 | 8.57E-05 | 83.17 | 6.29E-04 | 94.01 |
| MgF^+^ | 2.78E-07 | 5.18 | 8.01E-08 | 0.47 | 7.31E-07 | 7.72 | 2.41E-06 | 7.10 | 1.08E-06 | 5.25 | 4.95E-06 | 0.86 |  | 8.12E-05 | 14.43 | 3.06E-05 | 15.92 | 1.51E-05 | 14.70 | 2.58E-05 | 3.86 |
| CaF^+^ | 2.17E-08 | 0.40 | 2.18E-07 | 1.29 | 3.44E-08 | 0.36 | 9.11E-08 | 0.27 | 7.24E-08 | 0.35 | 2.72E-06 | 0.47 |  | 3.51E-06 | 0.62 | 8.09E-07 | 0.42 | 8.33E-07 | 0.81 | 5.95E-07 | 0.09 |
| NaF | 2.78E-09 | 0.05 | 5.89E-09 | 0.03 | 6.50E-09 | 0.07 | 1.18E-08 | 0.03 | 6.52E-09 | 0.03 | 2.14E-07 | 0.04 |  | 2.43E-05 | 4.31 | 3.87E-06 | 2.02 | 1.34E-06 | 1.30 | 1.38E-05 | 2.06 |
| HF | 1.51E-10 | 0.0028 | 2.19E-10 | 0.0013 | 4.71E-10 | 0.0050 | 1.26E-09 | 0.0037 | 7.50E-10 | 0.0037 | 1.83E-09 | 0.0003 |  | 1.65E-08 | 0.0029 | 1.13E-08 | 0.0060 | 2.51E-10 | 0.0002 | 1.87E-09 | 0.0003 |
| BF(OH)_3_^-^ | 9.91E-12 | 0.0002 | 5.27E-11 | 0.0003 | 2.25E-11 | 0.0002 | 1.32E-10 | 0.0004 | 2.05E-11 | 0.0001 | 3.56E-08 | 0.0062 |  | 7.35E-08 | 0.0131 | 1.24E-08 | 0.0065 | 1.88E-09 | 0.0018 | 7.18E-08 | 0.0107 |
| F complexed | 3.03E-07 | 5.64 | 3.04E-07 | 1.33 | 7.73E-07 | 8.16 | 2.52E-06 | 7.41 | 1.16E-06 | 5.64 | 7.92E-06 | 1.38 |  | 1.09E-04 | 19.38 | 3.53E-05 | 18.37 | 1.73E-05 | 16.81 | 4.03E-05 | 6.02 |
