# Supplementary material for: Geochemical processes controlling the groundwater chemistry and fluoride contamination in the Yuncheng Basin, China—An area with complex hydrogeochemical conditions
Source: PLoS One. 2018 Jul 26;13(7):e0199082. doi: 10.1371/journal.pone.0199082 (PMC6062146; doi:10.1371/journal.pone.0199082)
Supplement: S2 Table — (DOCX) [file pone.0199082.s002.docx]

S2 Table. General characteristics of groundwater samples

|  | sample | longitude | latitude | well depth （m） | T （℃） | pH | conductivity （µs） | HCO_3_^-^ （mg/l） | F (mg/l) | Cl (mg/l) | SO4 （mg/l） | NO3 （mg/l） | Ca （mg/l） | Mg（mg/l） | K （mg/l） | Na （mg/l） |
| --- | --- | --- | --- | --- | --- | --- | --- | --- | --- | --- | --- | --- | --- | --- | --- | --- |
|  |  |  |  |  |  |  |  |  |  |  |  |  |  |  |  |  |
| surface water | LY205 | 110º21´57.34" | 35º08´8.37" |  | 23.9 | 7.75 | 877 | 181.9 | 1.07 | 99.44 | 162.2 | 3.50 | 49.69 | 28.08 | 4.70 | 99.05 |
|  | YH04 | 110º50"33.34" | 34º55"26.34" |  | 18.5 | 7.80 | 12580 | 356.7 | 7.14 | 2687 | 4502 | 11.65 | 163.1 | 387.1 | 19.03 | 3105 |
|  | YH17 | 111º00"26.60" | 34º59"56.89" |  | 23.2 | 8.05 | 97900 | 661.0 | 15.4 | 52598 | 34487 | 163.6 | 25.05 | 765.0 | 31.20 | 49448 |
|  | YJ02 | 110º40´21" | 34º50´33" |  | 22.6 | 7.99 | 550 | 248.3 | 0.32 | 10.57 | 101 | 5.25 | 108.2 | 3.12 | 5.27 | 17.56 |
|  | YJ26 | 110º30´56" | 34º53´13" |  | 29.0 | 9.14 | 26600 | 1939 | 11.2 | 4409 | 7457 | 58.83 | 24.04 | 452.1 | 10.20 | 6292 |
|  | WX04 | 111º5´9" | 35º20´39" |  | 21.0 | 8.36 | 12580 | 559.6 | 3.94 | 208.9 | 230.3 | 6.76 | 60.12 | 60.00 | 3.24 | 274.6 |
|  | XX20 | 111º16"5.52" | 35º05´26.05" |  | 18.0 | 8.43 | 575 | 295.9 | 0.50 | 21.59 | 70.72 | 1.39 | 61.72 | 20.88 | 0.01 | 49.53 |
|  | XX07 | 111º18´59" | 35º14´06" |  | 5.1 | 8.48 | 880 | 251.2 | 0.50 | 10.99 | 89.19 | 19.95 | 79.00 | 19.77 | 2.24 | 21.08 |
| shallow groundwater | YJ06 | 110º17´19" | 34º46´41" | 30 | 19.0 | 7.20 | 1452 | 570.1 | 0.61 | 101.7 | 133.7 | 13.97 | 66.33 | 47.88 | 5.86 | 179.8 |
|  | YJ15 | 110º40´31.10" | 34º56´56.36" | 30 | 18.2 | 7.36 | 18370 | 693.2 | 10.5 | 3044 | 8295 | 27.94 | 280.5 | 489.6 | 1.18 | 4967 |
|  | YJ18 | 110º30´0.9" | 34º57´33.36" | 70 | 19.5 | 8.04 | 2020 | 811.4 | 4.34 | 110.9 | 279.8 | 13.79 | 9.01 | 26.40 | 1.05 | 455.8 |
|  | YJ29 | 110º19´43" | 34º55´57" | 40 | 18.0 | 7.68 | 218 | 265.8 | 3.06 | 279.7 | 311.1 | 3.98 | 35.07 | 60.72 | 1.75 | 275.8 |
|  | LY202 | 110º47´15.10" | 35º02´11.39" | 30 | 16.7 | 8.37 | 351 | 1053 | 9.42 | 272.2 | 790.0 | 13.52 | 13.02 | 43.68 | 1.04 | 858.1 |
|  | LY210 | 110º38´35.91" | 35º02´28.68" | 35 | 16.5 | 7.11 | 8760 | 538.6 | 3.62 | 1686 | 2812 | 14.82 | 124.2 | 349.9 | 3.45 | 1841 |
|  | LY211 | 110º31´46.70" | 35º01´16.32" | 50 | 18.1 | 8.56 | 1221 | 454.6 | 10.9 | 67.54 | 88.70 | 18.08 | 4.00 | 6.96 | 0.61 | 246.1 |
|  | WX05 | 111º20´42" | 35º30´5" | 45 | 18.9 | 7.91 | 859 | 467.9 | 1.28 | 16.52 | 38.11 | 5.41 | 18.03 | 36.24 | 1.56 | 117.1 |
|  | WX13 | 111º18´26" | 35º19´24" | 25 | 16.8 | 7.99 | 1101 | 291.7 | 2.99 | 39.01 | 45.80 | 9.23 | 10.02 | 18.00 | 0.30 | 114.8 |
|  | XX02 | 111º09´40.52" | 35º10´30.23" | 15 | 17.9 | 7.68 | 2460 | 148.3 | 1.45 | 102.1 | 203.3 | 13.98 | 35.87 | 88.20 | 14.08 | 8.28 |
|  | XX04 | 111º06´50.43" | 35º14´12.58" | 48 | 17.3 | 7.61 | 1942 | 273.5 | 0.60 | 187.1 | 318.2 | 11.71 | 60.12 | 85.20 | 3.12 | 149.0 |
|  | XX12 | 111º13´27.77" | 35º15´29.31" | 70 | 17.2 | 7.94 | 445 | 379.8 | 1.03 | 10.86 | 18.51 | 3.10 | 39.07 | 16.92 | 1.84 | 82.24 |
|  | YH02 | 110º52"57.15" | 34º56"57.63" | 70 | 17.3 | 8.52 | 6150 | 750.5 | 12.7 | 529.2 | 2213 | 26.56 | 22.44 | 72.96 | 1.67 | 1530 |
|  | YH03 | 110º50"26.40" | 34º54"3.65" | 30 | 18.8 | 7.76 | 1079 | 422.5 | 0.64 | 73.31 | 104.1 | 16.17 | 59.51 | 72.48 | 4.38 | 54.57 |
|  | YH12 | 110º58"45.4" | 35º16"19.37" | 65 | 23.4 | 8.15 | 1260 | 723.9 | 5.47 | 29.75 | 50.38 | 12.47 | 6.61 | 11.04 | 1.76 | 291.3 |
|  | YH27 | 110º57"6.33" | 34º55"56.71" | 10 | 17.0 | 7.29 | 899 | 363.0 | 0.53 | 37.23 | 78.00 | 10.49 | 121.8 | 20.40 | 3.79 | 21.58 |
|  | YH31 | 110º52"21.97" | 34º59"22.2" | 15 | 17.2 | 7.86 | 4820 | 609.9 | 5.33 | 644.5 | 1159 | 67.88 | 52.9 | 131.7 | 1.80 | 916.8 |
|  | YH41 | 110º58"40.74" | 35º02"52.76" | 9 | 17.8 | 7.70 | 4060 | 984.1 | 3.72 | 330.6 | 419.7 | 35.00 | 32.06 | 105.6 | 0.35 | 562.0 |
|  | 2014YJ-01 | 110º35´10" | 34º53´31" | 50 | 15.8 | 7.15 | 6490 | 451.5 | 1.27 | 1746 | 4108 | - | 428.9 | 605.2 | 0.76 | 1631 |
| Deep groundwater | YJ01 | 110º40´51" | 34º50´33" | 190 | 18.7 | 7.61 | 452 | 305.7 | 0.10 | 7.58 | 31.37 | 4.15 | 36.07 | 35.40 | 2.64 | 26.73 |
|  | YJ08 | 110º20´30" | 34º48´00" | 210 | 19.0 | 7.50 | 489 | 270.7 | 0.39 | 6.73 | 22.35 | 2.69 | 30.26 | 34.68 | 2.51 | 16.25 |
|  | YJ17 | 110º40´37.30" | 34º57´62.63" | 280 | 23.6 | 7.83 | 2690 | 437.9 | 0.53 | 308.1 | 837.2 | 8.13 | 106.8 | 166.4 | 3.72 | 329.0 |
|  | YJ19 | 110º30´0.9" | 34º57´33.36" | 280 | 21.3 | 7.92 | 3430 | 276.3 | 0.18 | 538.2 | 1035 | 7.70 | 130.9 | 106.0 | 5.96 | 597.1 |
|  | YJ20 | 110º22´54.73" | 34º59´38.07" | 120 | 19.5 | 8.25 | 1807 | 717.6 | 1.60 | 110.4 | 230.5 | 9.67 | 13.82 | 39.72 | 2.25 | 363.6 |
|  | YJ22 | 110º26´0.43" | 34º56´46.38" | 220 | 18.4 | 8.31 | 1897 | 831.0 | 2.05 | 96.76 | 216.4 | 18.61 | 12.02 | 28.08 | 1.83 | 418.3 |
|  | YJ27 | 110º21´15" | 34º54´46" | 120 | 19.4 | 8.10 | 110 | 517.6 | 1.20 | 36.23 | 105.8 | 2.22 | 21.84 | 18.84 | 0.97 | 208.7 |
|  | LY06 | 110º45´51.80" | 35º17´50.46" | 300 | 22.0 | 7.98 | 682 | 324.5 | 1.16 | 7.87 | 47.12 | 2.89 | 22.04 | 10.92 | 1.05 | 104.5 |
|  | LY07 | 110º34´42" | 35º13´39" | 200 | 20.0 | 7.79 | 81.6 | 505.0 | 0.93 | 7.99 | 29.67 | 4.88 | 16.03 | 24.36 | 3.20 | 147.0 |
|  | LY08 | 110º28´50" | 35º09´56" | 230 | 19.0 | 7.9 | 92.5 | 524.6 | 1.00 | 9.51 | 56.61 | 4.18 | 15.23 | 29.28 | 2.40 | 159.6 |
|  | LY09 | 110º25´54" | 35º07´0.72" | 280 | 22.0 | 7.92 | 98.4 | 549.1 | 1.24 | 10.64 | 79.73 | 4.71 | 16.03 | 24.12 | 10.05 | 183.75 |
|  | LY10 | 110º30´19" | 35º13´53" | 225 | 18.0 | 7.89 | 85.3 | 507.1 | 0.98 | 12.55 | 23.13 | 5.44 | 14.82 | 28.80 | 1.01 | 140.2 |
|  | LY13 | 110º38´55" | 35º11´23" | 240 | 19.0 | 8.04 | 1043 | 511.3 | 1.63 | 11.97 | 119.0 | 5.71 | 18.03 | 19.20 | 1.33 | 201.7 |
|  | LY14 | 110º34´22" | 35º10´37" | 280 | 23.0 | 8.16 | 917 | 507.8 | 1.67 | 10.92 | 48.85 | 7.78 | 16.03 | 22.68 | 1.80 | 163.4 |
|  | LY201 | 110º47´24.50" | 35º02´12.40" | 180 | 19.0 | 8.17 | 131.1 | 510.6 | 1.98 | 38.87 | 170.9 | 1.31 | 20.04 | 36.96 | 2.70 | 205.4 |
|  | LY203 | 110º46´43.14" | 35º04´5.69" | 110 | 18.2 | 8.30 | 127.7 | 587.5 | 3.15 | 27.42 | 177.1 | 1.17 | 17.43 | 30.12 | 3.09 | 245.6 |
|  | LY207 | 110º41´41.69" | 35º05´49.09" | 150 | 24.0 | 8.18 | 2100 | 293.8 | 1.45 | 291.6 | 411.1 | 7.93 | 48.89 | 41.76 | 3.46 | 362.4 |
|  | LY208 | 110º43´31" | 35º06´37" | 150 | 22.6 | 8.37 | 1436 | 494.5 | 2.09 | 66.75 | 230.1 | 8.99 | 30.86 | 24.84 | 2.89 | 260.7 |
|  | LY209 | 110º38´46.28" | 35º03´6.59" | 200 | 20.0 | 7.26 | 2260 | 345.5 | 1.15 | 220.3 | 660.7 | 5.79 | 64.12 | 70.08 | 6.72 | 381.3 |
|  | LY212 | 110º31´49.20" | 35º00´32.72" | 196.5 | 20.3 | 8.55 | 5930 | 307.8 | 1.95 | 1148 | 1933 | 5.86 | 186.3 | 261.6 | 8.38 | 1074 |
|  | WX03 | 111º04´54" | 35º20´37" | 130 | 21.2 | 7.71 | 835 | 433.0 | 0.91 | 31.93 | 59.14 | 7.30 | 21.82 | 48.85 | 2.56 | 97.4 |
|  | WX07 | 111º18´3" | 35º31´21" | 210 | 18.9 | 8.19 | 838 | 596.6 | 2.29 | 17.56 | 63.95 | 5.81 | 16.63 | 25.32 | 9.35 | 196.5 |
|  | WX08 | 111º10´57" | 35º26´50" | 180 | 23.0 | 8.12 | 1025 | 470.7 | 0.45 | 41.41 | 71.95 | 11.38 | 15.03 | 41.40 | 2.10 | 146.1 |
|  | WX10 | 111º17´54" | 35º26´43" | 85 | 16.5 | 7.97 | 984 | 216.8 | 0.97 | 29.13 | 42.52 | 4.83 | 17.23 | 45.12 | 1.49 | 16.73 |
|  | WX12 | 111º23´24" | 35º22´37" | 140 | 18.8 | 7.91 | 488 | 232.2 | 0.62 | 11.97 | 17.51 | 3.98 | 32.06 | 24.48 | 1.63 | 21.07 |
|  | WX15 | 111º18´48" | 35º19´26" | 200 | 18.9 | 7.55 | 6570 | 311.3 | 0.59 | 1402 | 2106 | 9.47 | 268.5 | 470.4 | 4.15 | 837.6 |
|  | WX16 | 111º9´49" | 35º21´5" | 245 | 20.0 | 8.10 | 1609 | 257.4 | 0.19 | 132.6 | 354.0 | 12.79 | 31.86 | 42.36 | 5.71 | 237.1 |
|  | WX17 | 111º10´43" | 35º20´33" | 90 | 19.0 | 8.44 | 1017 | 66.45 | 1.82 | 38.49 | 73.56 | 11.21 | 8.21 | 16.20 | 2.19 | 48.01 |
|  | XX01 | 111º09´53.73" | 35º10´36.11" | 250 | 18.0 | 8.42 | 772 | 244.8 | 1.72 | 63.24 | 101.3 | 2.96 | 29.85 | 14.52 | 1.24 | 120.4 |
|  | XX08 | 111º11´38.59" | 35º14´51.00" | 130 | 19.0 | 8.30 | 882 | 214.0 | 1.71 | 12.01 | 55.66 | 5.13 | 12.62 | 23.04 | 1.37 | 58.10 |
|  | XX10 | 111º16´16.92" | 35º16´38.96" | 280 | 23.3 | 8.24 | 789 | 208.4 | 0.89 | 74.00 | 114.6 | 1.76 | 29.05 | 23.16 | 1.63 | 103.9 |
|  | YH01 | 110º53"13.33" | 34º56"22.37" | 350 | 24.7 | 8.25 | 1780 | 398.0 | 1.29 | 195.7 | 347.2 | 8.13 | 27.45 | 36.84 | 1.75 | 343.8 |
|  | YH07 | 110º48"40.22" | 34º53"29.27" | 115 | 23.3 | 8.11 | 603 | 223.1 | 0.85 | 11.39 | 134.2 | 4.03 | 66.93 | 16.32 | 2.09 | 48.29 |
|  | YH16 | 110º01"3.17" | 34º57"32.9" | 100 | 18.6 | 7.56 | 1240 | 270.0 | 0.40 | 53.15 | 144.0 | 8.28 | 73.14 | 18.72 | 4.12 | 86.44 |
|  | YH20 | 111º07"39.29" | 35º00"34.27" | 100 | 19.7 | 8.16 | 1087 | 271.4 | 1.51 | 156.6 | 126.5 | 2.83 | 54.90 | 30.60 | 3.83 | 142.3 |
|  | YH23 | 111º03"32.68" | 35º07"26.78" | 262 | 19.9 | 8.06 | 1204 | 601.5 | 1.49 | 49.86 | 96.00 | 11.44 | 26.45 | 32.88 | 1.37 | 215.8 |
|  | YH26 | 111º02"22.70" | 35º04"13.11" | 300 | 21.6 | 8.15 | 1702 | 248.3 | 1.22 | 173.4 | 470.9 | 6.60 | 56.31 | 48.84 | 1.58 | 275.9 |
|  | YH28 | 110º57"52.31" | 34º55"38.97" | 180 | 20.1 | 7.80 | 784 | 242.0 | 0.49 | 50.75 | 144.4 | 3.77 | 96.79 | 14.52 | 4.28 | 53.58 |
|  | YH30 | 110º47"55.25" | 35º01"41.42" | 220 | 18.2 | 8.36 | 1397 | 524.6 | 1.03 | 102.3 | 280.2 | 5.79 | 25.45 | 67.20 | 3.21 | 242.0 |
|  | YH32 | 110º54"1.91" | 34º59"24.39" | 340 | 21.6 | 8.05 | 1267 | 460.2 | 1.17 | 81.74 | 210.7 | 7.11 | 28.05 | 51.24 | 1.84 | 199.7 |
|  | YH35 | 110º55"34.46" | 35º10"37.84" | 120 | 16.5 | 7.90 | 1545 | 670.1 | 2.28 | 104.1 | 146.0 | 16.32 | 12.22 | 45.96 | 2.75 | 293.4 |
|  | YH39 | 111º00"42.00" | 35º00"58.05" | 320 | 21.0 | 7.98 | 2080 | 280.5 | 1.19 | 303.5 | 394.8 | 23.96 | 69.73 | 66.00 | 2.11 | 294.3 |
